# Reproduction disrupts stem cell homeostasis in testes of aged male *Drosophila* via an induced microenvironment

**DOI:** 10.1371/journal.pgen.1008062

**Published:** 2019-07-11

**Authors:** Yi Chieh Chang, Hsin Tu, Jing-Yi Chen, Ching-Chin Chang, Shu Yuan Yang, Haiwei Pi

**Affiliations:** 1 Graduate Institute of Biomedical Sciences, College of Medicine, Chang Gung University, Taoyuan, Taiwan; 2 Department of Biomedical Sciences, College of Medicine, Chang Gung University, Taoyuan, Taiwan; 3 Craniofacial Research Center, Taoyuan Chang Gang Memorial Hospital, Taoyuan, Taiwan; 4 Molecular Medicine Center, College of Medicine, Chang Gung University, Taoyuan, Taiwan; 5 Department of Pathology, Chang Gung Memorial Hospital, Taoyuan, Taiwan; College de France CNRS, FRANCE

## Abstract

Stem cells rely on instructive cues from their environment. Alterations in microenvironments might contribute to tissue dysfunction and disease pathogenesis. Germline stem cells (GSCs) and cyst stem cells (CySC) in *Drosophila* testes are normally maintained in the apical area by the testicular hub. In this study, we found that reproduction leads to accumulation of early differentiating daughters of CySCs and GSCs in the testes of aged male flies, due to hyperactivation of Jun-N-terminal kinase (JNK) signaling to maintain self-renewal gene expression in the differentiating cyst cells. JNK activity is normally required to maintain CySCs in the apical niche. A muscle sheath surrounds the *Drosophila* testis to maintain its long coiled structure. Importantly, reproduction triggers accumulation of the tumor necrosis factor (TNF) Eiger in the testis muscle to activate JNK signaling via the TNF receptor Grindelwald in the cyst cells. Reducing Eiger activity in the testis muscle sheath suppressed reproduction-induced differentiation defects, but had little effect on testis homeostasis of unmated males. Our results reveal that reproduction in males provokes a dramatic shift in the testicular microenvironment, which impairs tissue homeostasis and spermatogenesis in the testes.

## Author summary

Proper differentiation of stem cell progeny is necessary for preservation of tissue homeostasis. In *Drosophila* testes, somatic cyst cells derived from the cyst stem cells (CySCs) control the differentiation of the neighboring germ cells. Disruption of CySC daughter cyst cell differentiation leads to failure in sperm production. Interestingly, we found that reproduction triggers hyperactivation of Jun-N-terminal kinase (JNK) signaling to sustain CySC self-renewal gene expression in differentiating cyst cells, leading to accumulation of immature cyst cell and germ cells at the expense of mature cells in the testes of aged males. Endogenous JNK signaling is also required for CySC maintenance. Moreover, we found that the JNK signaling is hyperactivated via reproduction-induced accumulation of tumor necrosis factor (TNF) in testicular smooth muscle that surrounds the testis to support its long coiled structure. The reproduction-induced phenotypes were only observed in the testes of aged and mated males, but not in testes form young mated males or aged unmated males, indicating that it is a combined effect of reproduction and aging. Our results reveal that reproduction impedes sperm production in aged males, and identify testicular muscle as an inducible signaling center for spermatogenesis in *Drosophila*.

## Introduction

The adult mammalian testis generates millions of mature sperm every day [1, 2]. Spermatogenesis begins when germline stem cell (GSC) progeny enter differentiation rather than remaining in a self-renewal state [3]. GSCs are maintained by secreted factors from the surrounding testicular niche, which is composed of different types of somatic cell populations in different species [4, 5]. Failure by GSCs to balance self-renewal and differentiation impedes sperm production and consequently reproduction. In mammalian seminiferous tubules, Sertoli cells which are associated with the developing germ cells are the key contributors to the GSC niche [5]. In *Drosophila* testes, cyst stem cells (CySCs) are located in close proximity to the GSCs and are required to maintain GSC self-renewal and prevent differentiation [6–9]. Additionally, a cluster of 10–15 somatic cells located at the apical tip of the *Drosophila* testis, the hub, is crucial for self-renewal of both the GSCs and CySCs that surround it [10, 11]. Division of a GSC or CySC generates a daughter stem cell that remains close to the hub, thereby maintaining the stem cell fate, while the other daughter cell is displaced from the hub to initiate differentiation [10–13]. Consequently, both GSCs and CySCs are restricted to the apical region, generating an apical-to-basal gradient of germ cell differentiation.

A sheath of smooth muscles surrounds both the mammalian seminiferous tubules and the *Drosophila* testes [10, 14, 15]. Contraction of this peritubular smooth muscle facilitates the transport of sperm and testicular fluid in the seminiferous tubules [16, 17]. The peritubular cells also modulate the functions of Sertoli cells and GSCs via paracrine signaling, thereby acting as an additional niche in the mammalian testis [5, 18, 19]. In *Drosophila*, the testis sheath, which is composed of smooth muscles and the pigment cells, appears to be essential for the long coiled structure of the testes [20, 21]. Mutations that affect sheath development result in smaller or abnormally-shaped testes [22, 23]. However, in these cases, germ cells and somatic cells (including hub cells and cyst cells) are still observed in the apical region, suggesting that unlike mammalian peritubular cells, the testis sheath is not essential for early spermatogenesis in *Drosophila* [22].

It has been observed in diverse organisms that reproduction is associated with a reduction in future fertility [24–26]. In this study, we found that reproduction induces ectopic expression of the self-renewal protein Zfh-1 in the cyst lineage cells of aged testes. As a result, cyst cell differentiation is disrupted and CySC-like cells over-proliferate, leading to overproduction and accumulation of undifferentiated germ cells away from the hub. We found that the tumor necrosis factor-Jun N-terminal kinase (TNF-JNK) signaling pathway is hyperactivated and that genetic reduction of TNF-JNK signaling activity suppresses these defects. Interestingly, reproduction induces substantial accumulation of the TNF superfamily ligand Eiger in the testis muscle that activates TNF receptor Grindelwald (Grnd), which is specifically expressed in cyst cells following reproduction. Our study uncovers a cost of reproduction through ectopic stem cell self-renewal during spermatogenesis, and also reveals unexpected conservation from flies to humans of the role of smooth muscle as a testicular signaling center to influence spermatogenesis.

## Results

### Reproduction induces overproduction of immature cells away from the testicular hub in the testes of aged male flies

**Fig 1:**
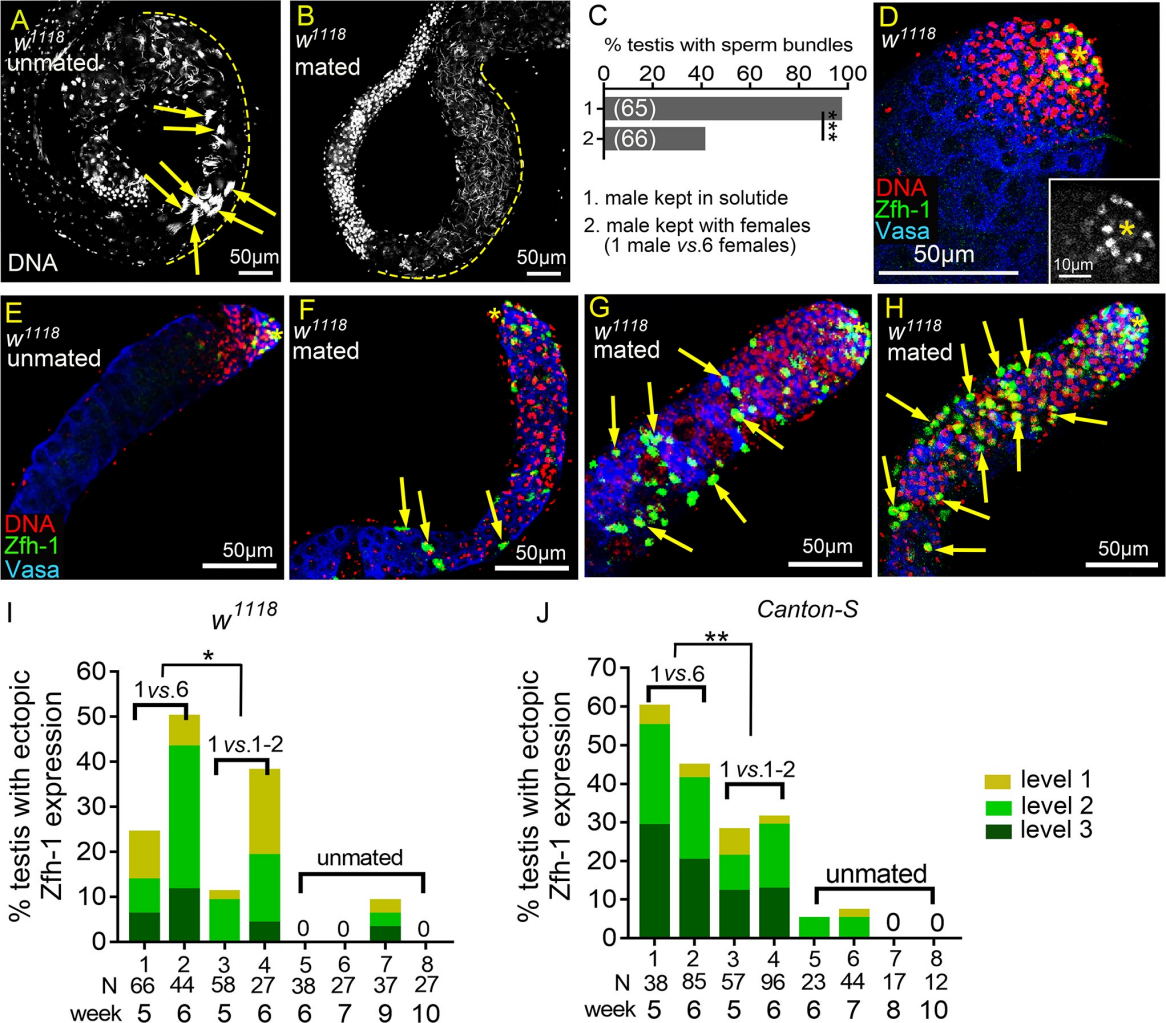
Reproduction induces ectopic Zfh-1 expression away from the hub. (A and B) Basal ends of 5w-old *w*^*1118*^ testes stained for DNA to visualize the bundles of spermatid nuclei (Hoescht 33342). (A) Testis from males kept in solitude. Sperm bundles are indicated by arrows. (B) Testis from a mated male (1 male *vs*. 6 females). There was no sperm bundle at the basal end. (C) Percentages of the testes containing at least one sperm bundle. (D-H) Testes from *w*^*1118*^ males immunostained for Zfh-1 (CySC/early cyst cell, green), co-stained for Vasa (germ cells, blue) and DNA (nuclei, red). Asterisks indicate testis hubs. (D) Apical region of a 1-3-day-old testis. Zfh-1-positive cells and small, DNA-bright Vasa-positive early germ cells were only present in the apical region. Inset shows the Zfh-1 expression in the tip. (E-H) 5w-old testes from the males kept in solitude (unmated) (E), or from single males mated with six females (1 *vs*. 6) (F-H). Ectopic Zfh-1-positive cells are indicated by arrows in the testes exhibiting level 1 (F), level 2 (G) and level 3 (H) ectopic Zfh-1 expression. In level 2 (G) and level 3 (H) testes, ectopic Zfh-1-positive cells were usually associated with small germ cells. (I and J) Percentages of *w*^*1118*^ (I) and *Canton-S* (J) testes with ectopic Zfh-1 expression. Extra Zfh-1-positive cells (levels 1–3) were observed in testes from single male mated with 6 females (1 *vs*. 6) and 1–2 males (1 *vs*. 1–2) but rarely seen in the testes from unmated males. The asterisks indicate the significance comparing total testes from week-5 and -6 males in 1 *vs*. 6 to 1 *vs*. 1–2 mating ratio. N = number of testes scored. P-values shown in C, I, and J were calculated with Chi-squared test. *p<0.05, **p<0.01, and ***p<0.001.

A previous study has shown that the 34-day-old male flies are nearly sterile after mating with two females every two days, whereas 34-day-old males not previously mated exhibited high fecundity [27]. To investigate whether reproduction influences spermatogenesis in aged male flies, we supplied single males with six virgin females every week for 5 or 6 weeks and then assessed the presence of sperm bundles at the basal ends of their testes [28]. Sperm bundles were formed in 97% (N = 65) of the testes of six and seven week-old control males that had been kept in solitude without females (Fig 1A and column 1 in Fig 1C). For males supplied with females, only 41% (N = 66) of their testes had sperm bundles present at the basal end (Fig 1B and column 2 in Fig 1C), suggesting defective spermatogenesis in mated males.

Spermatogenesis starts with the division of GSCs to generate the primary spermatogonia, the goniablasts (Gbs). Gbs undergo transit-amplifying divisions to ultimately give rise to cysts containing 16 interconnected spermatogonia [29]. The early germ cells including GSCs, Gbs and spermatogonia are normally localized in the apical region of the testis, recognizable by their smaller cell size and the intensely DNA-staining nuclei (Fig 1D). Somatic cyst cells tightly control germ cell differentiation [30–33]. CySCs and early cyst cells are also located in the apical region and can be identified by expression of Zinc finger homeodomain-1 (Zfh-1) protein, which is expressed at high levels in CySCs and in progressively lower levels in early cyst cells enclosing the Gbs and the 2-, 4-, and 8-cell spermatogonia clusters [6] (Fig 1D and inset). Similar to what has been found for testes from aged male flies described in previous studies [34, 35], we observed testicular involution in 5 week-old (5w-old) control males kept in solitude (unmated), accompanied by a decrease in the number of spermatogonia and an approximately 50% reduction in the number of Zfh-1-positive cells [from 34±4.6 (N = 45) for 1-3-day old males to 19±4.2 (N = 66) for 5w-old males] (Fig 1E). The most obvious difference in testes between these unmated control males and the males mated with six females each week for 5 weeks was the presence of ectopic Zfh-1-positive cells away from the testis tip (Fig 1F–1H). In some cases, numerous Zfh-1-positive cells were found throughout the testes of mated males, suggesting overproduction of Zfh-1-positive cells (Fig 1H). We also observed accumulation of early germ cells accompanied by the ectopic Zfh-1-positive cells (Fig 1F–1H). We classified the ectopic Zfh-1 expression in the testes of mated males into three categories (levels 1–3) based on the numbers and location of Zfh-1-positive cells. Testes with more than 200 Zfh-1-positive cells, representing a more than 10-fold increase compared to the testes of aged unmated males, were scored as “level 3” (Fig 1H), whereas those with 50–200 Zfh-1-positive cells were scored as “level 2” (Fig 1G). Testes scored as “level 1” had fewer than 50 Zfh-1-positive cells but also exhibited mis-localization of Zfh-1-positive cells away from the tip (arrows in Fig 1F). We found that 26% (N = 66) and 50% (N = 44) of testes, respectively, from 5w- and 6w-old males mated with six virgin females every week exhibited ectopic Zfh-1 expression (columns 1 and 2 in Fig 1I). In contrast, ectopic Zfh-1-positive cells were never observed in the testes of 6w- and 7w-old unmated males (columns 5 and 6 in Fig 1I). Ectopic Zfh-1-positive cells was also observed in mated *Canton-S* flies; on average 50% of the testes from 5w- and 6w-old mated *Canton-S* males had ectopic and extra Zfh-1-positive cells (columns 1 and 2 in Fig 1J). Only a few testes (4/67) from 6w- and 7w-old unmated *Canton-S* males had ectopic Zfh-1-positive cells (columns 5 and 6 in Fig 1J).

Reproduction reduces the lifespan of male flies [24]. Since we found that the survival rate of 5w-old mated males was much lower than that of 5w-old unmated males (S1 Fig), it is possible that the ectopic Zfh-1-positive cells in testes of mated males is the result of accelerated aging. To test whether aging is the major causative factor of the ectopic Zfh-1 phenotype, we examined testes from unmated 9w- and 10w-old males (i.e. when survival rates declined rapidly to those comparable to 4w- and 5w-old mated flies, respectively) (N = 99) (S1 Fig). Ectopic Zfh-1-positive cells were observed in only 8% of the testes of 9w-old unmated males, and none of the testes of 10w-old unmated males had the ectopic cells (columns 7 and 8 in Fig 1I). Similarly, none of the testes of 8w- and 10w-old *Canton-S* unmated males exhibited ectopic Zfh-1 expression (columns 7 and 8 in Fig 1J). Our results suggest that accelerated aging is unlikely to be solely responsible for the reproduction-induced phenotypes we observed in the testes of mated male flies.

**Fig 2:**
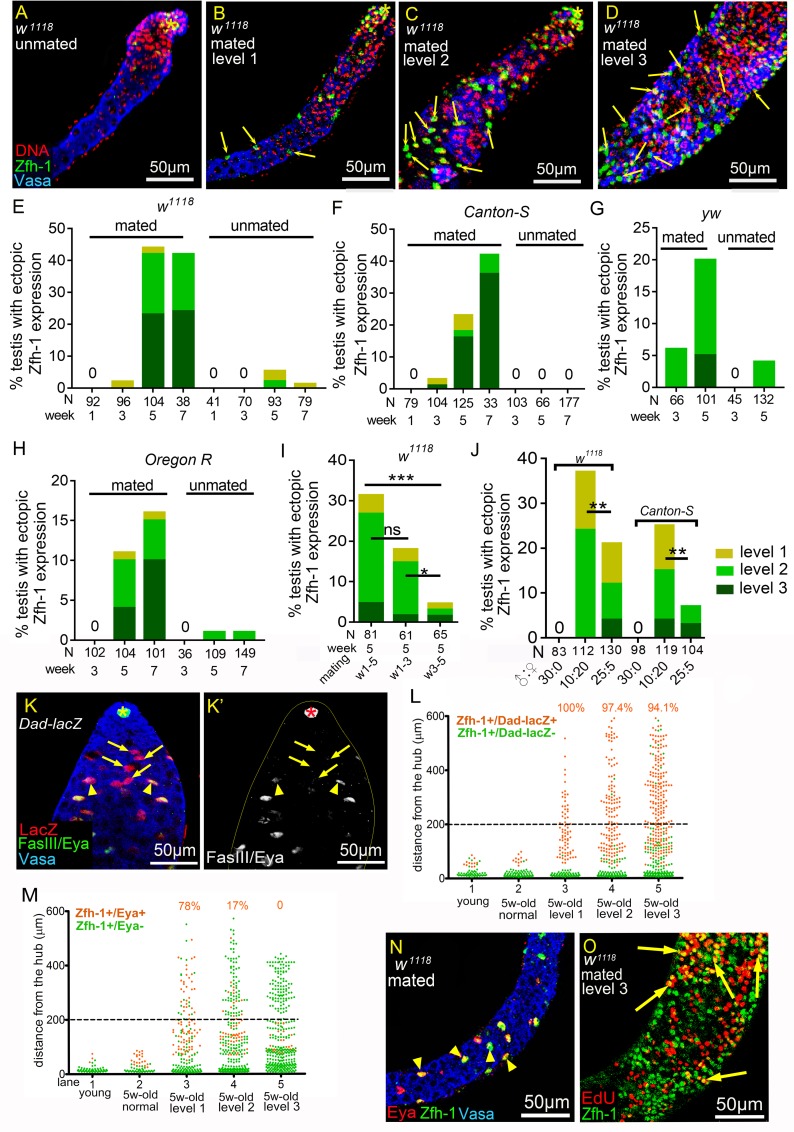
Reproduction disrupts cyst cell differentiation. (A-D) 5w-old *w*^*1118*^ testes immunostained for Zfh-1 (green), co-stained for Vasa (blue), and DNA (red). Asterisks mark the hubs. Arrows indicate ectopic Zfh-1-positive cells. (A) Normal testis from an unmated male (30 *vs*. 0). Zfh-1-positive cells and early germ cells were located near the hub. (B-D) Testes from mated *w*^*1118*^ males (10 *vs*.20), exhibiting ectopic Zfh-1expression at level 1 (B), level 2 (C), and level 3 (D). (E-H) Percentages of testes with ectopic Zfh-1-positive cells from the males at different ages. Numbers of the testes scored are shown in the parentheses. (E-H) Testes from mass-mated (10 *vs*. 20) or unmated (30 *vs*. 0) males of *w*^*1118*^ (E), *Canton-S* (F), *yw* (G), and *Oregon R* (H) at different ages. (I) Testes from 5w-old *w*^*1118*^ males mated with females (10 *vs*. 20) for five weeks (week 1–5), the first three weeks (week 1–3), or the last three weeks (week 3–5). (J) Testes from 4w-old *w*^*1118*^ and *Canton-S* males mated to females with the ratios of 30:0, 10:20, and 25:5. P-values in I and J were calculated with Chi-squared test. ns: *p*>0.05, *p<0.05, **p<0.01, and ***p<0.001. (K and K’) 3-day-old *Dad-lacZ* testis immunostained for β-galactosidase (red in K), co-stained for FasIII and Eya (green in K, and white in K’) and Vasa (blue in K). Arrows indicate the LacZ-positive and Eya-negative cyst cells. Arrowheads mark the cells double positive for LacZ and Eya. Asterisks indicate hubs. (L and M) Graphs showing the distance of Zfh-1-positive cells to the hub in the mated testes from *Dad-lacZ* (L) and *w*^*1118*^ males (M). Each dot indicates a single Zfh-1-positive cell. Ectopic Zfh-1-positive cells are defined as cells 200 μm away from the hub (dash line). Percentages of the double positive cells among the total ectopic Zfh-1-positive cells are shown at the top of each column. (N) A testis from mated 5w-old *w*^*1118*^ exhibiting ectopic Zfh-1 expression in differentiated cyst cells. Arrowheads mark the ectopically expressed Zfh-1 (green) in Eya (red)-positive cells away from the hub (large, Vasa-positive cells). (O) EdU labeling of a testis displaying level 3 ectopic Zfh-1 expression from mated 5w-old *w*^*1118*^ males. The arrows indicate the double-positive cells for EdU (red) and Zfh-1 (green) away from the hub. Mass-mating schemes were conducted for all experiments.

To further characterize the phenotypes in a larger sample size, we assessed the effects of reproduction on spermatogenesis using a mass-mating scheme. Male flies of different genetic backgrounds (*w*^*1118*^, *Canton-S*, *yw* and *Oregon R*) were examined. We found that when 10 males were supplied with 20 virgin females every week, they also exhibited age-dependent ectopic Zfh-1 expression in 44% (N = 104), 23% (N = 125), 20% (N = 101) and 11% (N = 104) of the testes, respectively, from mated 5w-old *w*^*1118*^, *Canton-S*, *yw*, and *Oregon R* flies (Fig 2B–2H). The differences in the percentages of the ectopic Zfh-1 expression among these strains might not be due to differential mating frequencies, since we found that the young males of *w*^*1118*^, *CS* and *OR* strains would mate to females of comparable numbers within 24 hours (S2 Fig). None or very few testes of 1w- and 3w-old mated males possessed ectopic Zfh-1-positive cells (Fig 2E–2H). Ectopic Zfh-1 expression was rarely observed in the testes of unmated males (N = 30), regardless of age and genetic backgrounds (Fig 2A and 2E–2H). Interestingly, 5w-old males that had mated from weeks 1 to 3 exhibited more level-2 and level-3 Zfh-1 phenotypes than those that had mated from weeks 3 to 5 (Fig 2I), suggesting that reproduction in early life has a stronger effect to induce ectopic Zfh-1 phenotypes.

We evaluated the possible correlation between reproductive rates and extra Zfh-1 cells by varying the number of females available for mating. When we reduced the mating system to 1–2 females per isolated male each week (Fig 1I and 1J), on average 20% of testes of *w*^*1118*^ mated males (N = 85) and 30% of those of *Canton-S* mated males (N = 153) at 5w- and 6w-old also showed ectopic Zfh-1 expression, although this was a significantly lower proportion than exhibited by males mated with six females (Fig 1I and 1J). Consistently, we observed lower percentages of extra Zfh-1-positive cells in the testes of male *w*^*1118*^ and *Canton-S* flies mated with females in a 5:1 ratio (25 males *versus* five females) than for males mated at a 1:2 ratio (10 males *versus* 20 females) (Fig 2J).

These results indicate that reproduction can lead to ectopic Zfh-1 expression in the testes of aged male flies and that the levels are correlated with reproductive rate. Since the testes of males in mass mating exhibited reproduction-induced phenotypes similar to those in single-male mating, we conducted mass-mating experiments in most of the following studies to achieve larger samples sizes.

### Reproduction induces an accumulation of early germ cells by Dpp-dependent BMP signaling activation

Zfh-1 maintains CySCs by preventing them from entering differentiation [6]. To examine whether cyst cell differentiation was blocked in the ectopic Zfh-1-positive cells, we assessed expression of *Dad-lacZ* [a *lacZ* enhancer trap line for *Daughter against dpp* (*Dad*) [36]] and Eyes absent (Eya). *Dad* is a bone morphogenetic protein (BMP) signaling target [36], and *Dad-lacZ* expression was normally initiated in early differentiating cyst cells associated with four- to eight-cell spermatogonia clusters (arrows Fig 2K and 2K’). Eya was expressed later in the more mature cyst cells associated with late spermatogonia (arrowheads in Fig 2K and 2K’). In testes of mated males exhibiting ectopic Zfh-1 expression at level 1, most of the ectopic Zfh-1-positive cells were also positive for *Dad-lacZ* (100%) and Eya (78%) (column 3 in Fig 2L and 2M). In testes exhibiting level 2 and level 3 phenotypes, Eya expression was blocked in many of the ectopic Zfh-1-positive cells (83% and 100% for level 2 and level 3 phenotypes, respectively, Fig 2M), whereas *Dad-lacZ* expression was unaffected (Fig 2L). We even observed Zfh-1 expression in Eya-positive cells distant from the hub (arrowheads in Fig 2N), suggesting that differentiation had not yet been severely disrupted. These data indicate that most of the ectopic Zfh-1-positive cells in testes exhibiting level 2 and level 3 phenotypes had initiated cell differentiation but were unable to proceed to stages when Eya is activated. Only CySCs actively divide in the cyst cell lineage [37]. In testes scored as level 3, some ectopic Zfh-1-positive cells proceeded through the S phase and incorporated EdU (arrows in Fig 2O), suggesting that reproduction may lead to the induction and/or maintenance of CySC characteristics away from the hub.

**Fig 3:**
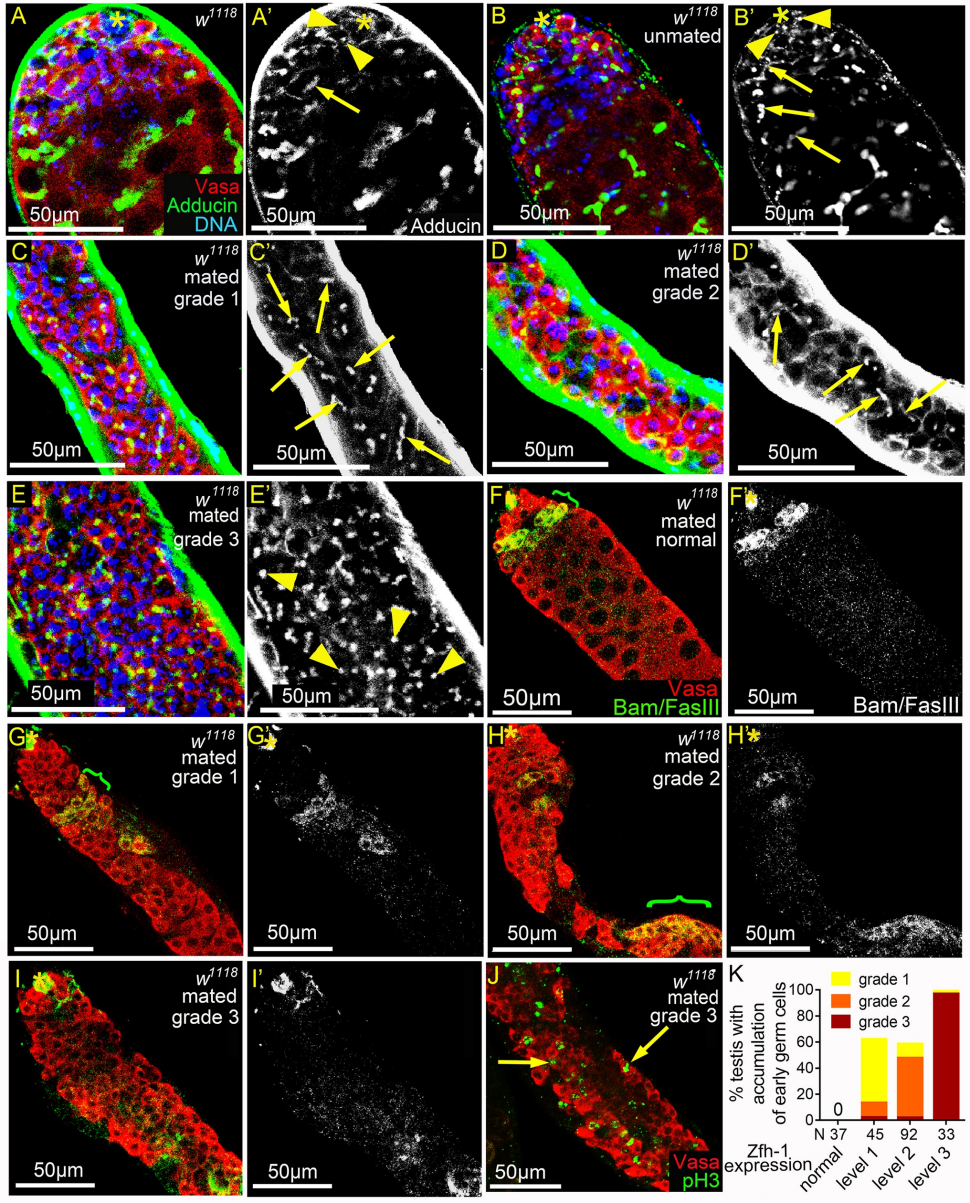
Reproduction leads to early germ cell accumulation and reduced Bam expression. (A-E’) *w*^*1118*^ testes immunostained for Adducin (green, white in A’-E’), co-stained for Vasa (red) and DNA (blue). Dot spectrosomes and thin fusomes are indicated by arrowheads and arrows, respectively. Asterisks mark the hubs. (A-B’) Dot spectrosomes and thin fusomes were observed only in the apical region of the testes from 1-3-day-old (A and A’) and 5w-old unmated (B and B’) males. (C-D’) Thin fusomes were found in the middle part of the grade 1 (arrows in C and C’) and grade 2 (arrows D and D’) testes that exhibit excess early germ cells. (E and E’) Dot-like spectrosomes (arrowheads) were observed away from the hub in the grade 3 testes. (F-I’) Testes from mated 5w-old *w*^*1118*^ males immunostained for Bam and FasIII (green in F-I and white in F’-I’), co-stained for Vasa (red). Bam-positive regions are indicated with brackets. Asterisks mark the hubs. (G-I’) Testes with expansion of early germ cells. Bam expression was markedly reduced and the onset of Bam expression was delayed in grade 1 and 2 testes (G and H). Anti-Bam signals were not detected in grade 3 testes (I). (J) Testis from mated 5w-old *w*^*1118*^ male immunostained for pH3 (green), co-stained for Vasa (red). Arrows indicate the single germ cells undergoing mitosis. (K) Percentages of mated 5w-old *w*^*1118*^ testes exhibiting excess small germ cells. The percentages and severity of accumulation of early germ cells (Y-axis) were positively correlated with the degrees of ectopic Zfh-1 expression (X-axis). Mass-mating of 10 males and 20 females was conducted for all experiments.

Signals from the cyst cell lineage are critical for GSC self-renewal and for differentiation of spermatogonia into spermatocytes [6–8, 30–32, 38–41]. We found that germ cell differentiation was severely disrupted in the majority of testes exhibiting ectopic Zfh-1-positive cells, as demonstrated by the accumulation of extra early germ cells at the expense of spermatocytes (Fig 1G and 1H, and Figs 2C, 1D and 3K). We categorized the phenotype of early germ cell accumulation into three grades (1, 2, and 3) (see the Methods section). In contrast to 1-3-day old and unmated 5w-old males whose early germ cells are restricted to the apical region of the testes (Fig 3A–3B’) [35], testes categorized as grade 1 and 2 exhibited expansion of early germ cells, most of which were spermatogonia as evidenced by the presence of thin, branched fusomes (arrows in Fig 3C–3D’) [42]. Testes categorized as grade 3 were full of early germ cells, some of which contained spectrosomes that are characteristic of GSCs and Gbs [43] (Fig 3E and arrowheads in Fig 3E’). Bag of marbles (Bam) protein accumulation controls timing of the switch from spermatogonia proliferation to differentiation [43–46]. In 5w-old unmated males, we detected Bam in the 4–16 cell stages of spermatogonia in the apical region (Fig 3F and 3F’) [44]. Onset of Bam accumulation appeared to be delayed or Bam levels were markedly reduced in testes of grades 1 and 2 (Fig 3G–3H’). Anti-Bam immunostaining generated almost no signal in grade 3 testes (Fig 3I and 3I’). Thus, Bam expression is suppressed in the testes of mated males exhibiting early germ cell expansion. Single germ cells positive for the mitosis marker phospho-Histone H3 (pH3) were also observed away from the hub in grade 3 testes (arrows in Fig 3J), suggesting that GSC- or Gb-like cells are present in grade 3 testes.

**Fig 4:**
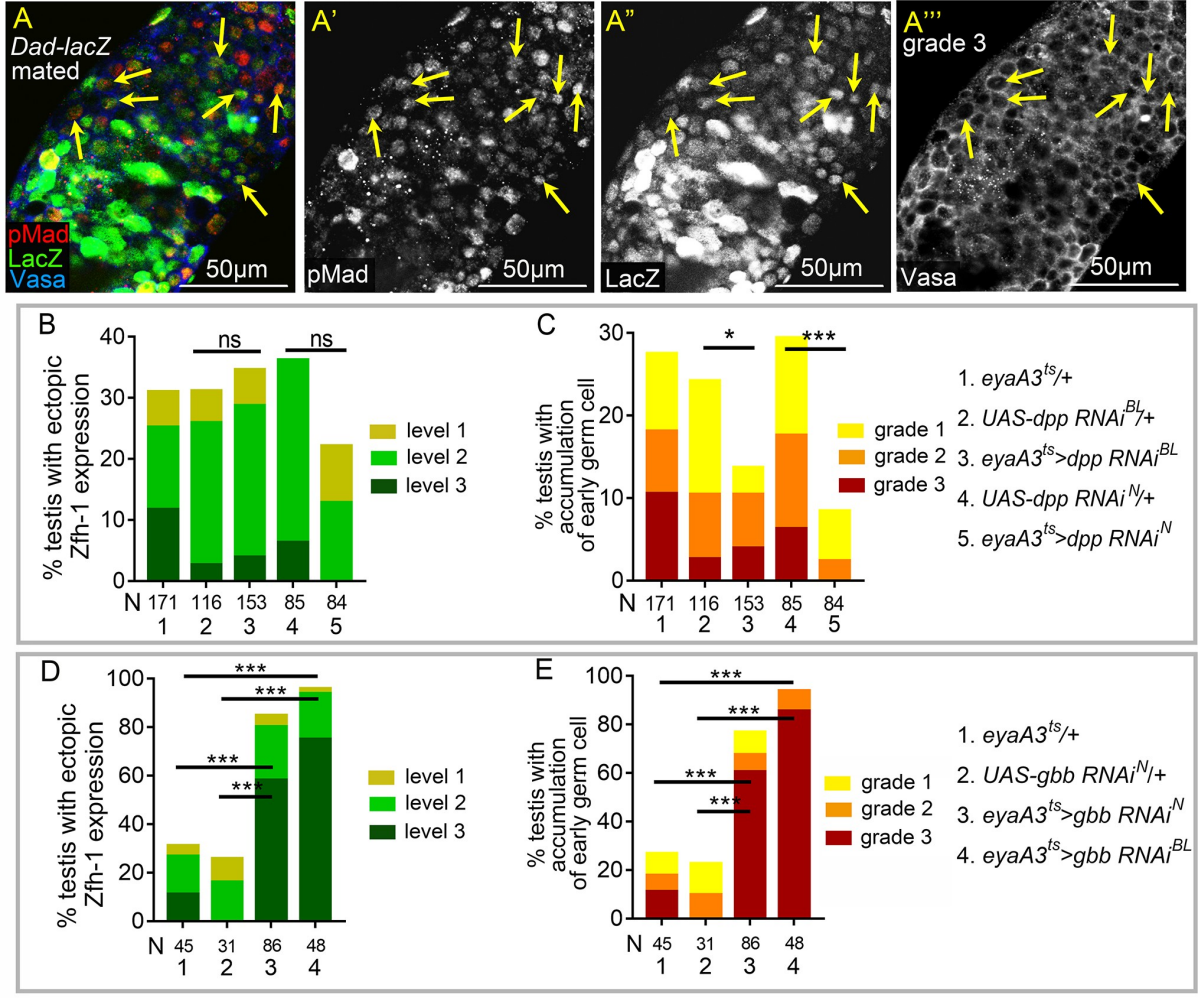
*dpp*-dependent BMP signaling activation in germ cells by reproduction. (A-A”‘) A grade 3 *Dad-lacZ* testis from mated male immunostained for pMad (red in A, and white in A’), co-stained for β-galactosidase (green in A, and white in A”), and Vasa (blue in A, and white in A’”). Arrows indicate the small germ cells positive for both pMad and LacZ. (B and C) Knockdown of *dpp* in cyst lineage cells decreased the percentages of germ cell accumulation (C), but did not significantly suppress ectopic Zfh-1expression (B). (D and E) Reducing *gbb* from cyst cell lineage failed to suppress ectopic Zfh-1 expression (D) and expansion of early germ cells (E) in mated testes. All p-values were calculated with Chi-squared test. ns: p>0.05, *p<0.05, **p<0.01, and ***p<0.001. Mass-mating was conducted for all experiments.

Apart from its activity in cyst cells, BMP signaling is activated in GSCs to maintain self-renewal and to suppress *bam* transcription [43, 47]. We wondered whether BMP signaling is hyperactivated in the testes of mated males. Examination of phosphorylated Mad (pMad), the activated *Drosophila* Smad protein and *Dad-lacZ* revealed numerous ectopic pMad-positive and *Dad-lacZ-*positive germ cells away from the hub in grade 3 testes (arrows in Fig 4A–4A”‘). The BMP ligands Decapentaplegic (Dpp) and Glass bottom boat (Gbb) are expressed in early cyst cells and the hub cells [43]. Interestingly, depletion of Dpp or Gbb from cyst lineage cells by RNA interference showed that *dpp* is required for undifferentiated germ cell expansion in the testes of mated males and that *gbb* is dispensable for this phenotype (Fig 4C and 4E). Unlike the marked suppression of early germ cell expansion, knockdown of *dpp* or *gbb* did not significantly suppress ectopic Zfh-1 expression in testes of mated males (Fig 4B and 4D). Therefore, our results show that Dpp-mediated BMP signaling hyperactivation via reproduction leads to delayed or blocked early germ cells differentiation.

### JNK signaling is hyperactivated in the somatic cyst cells in testes of mated males

**Fig 5:**
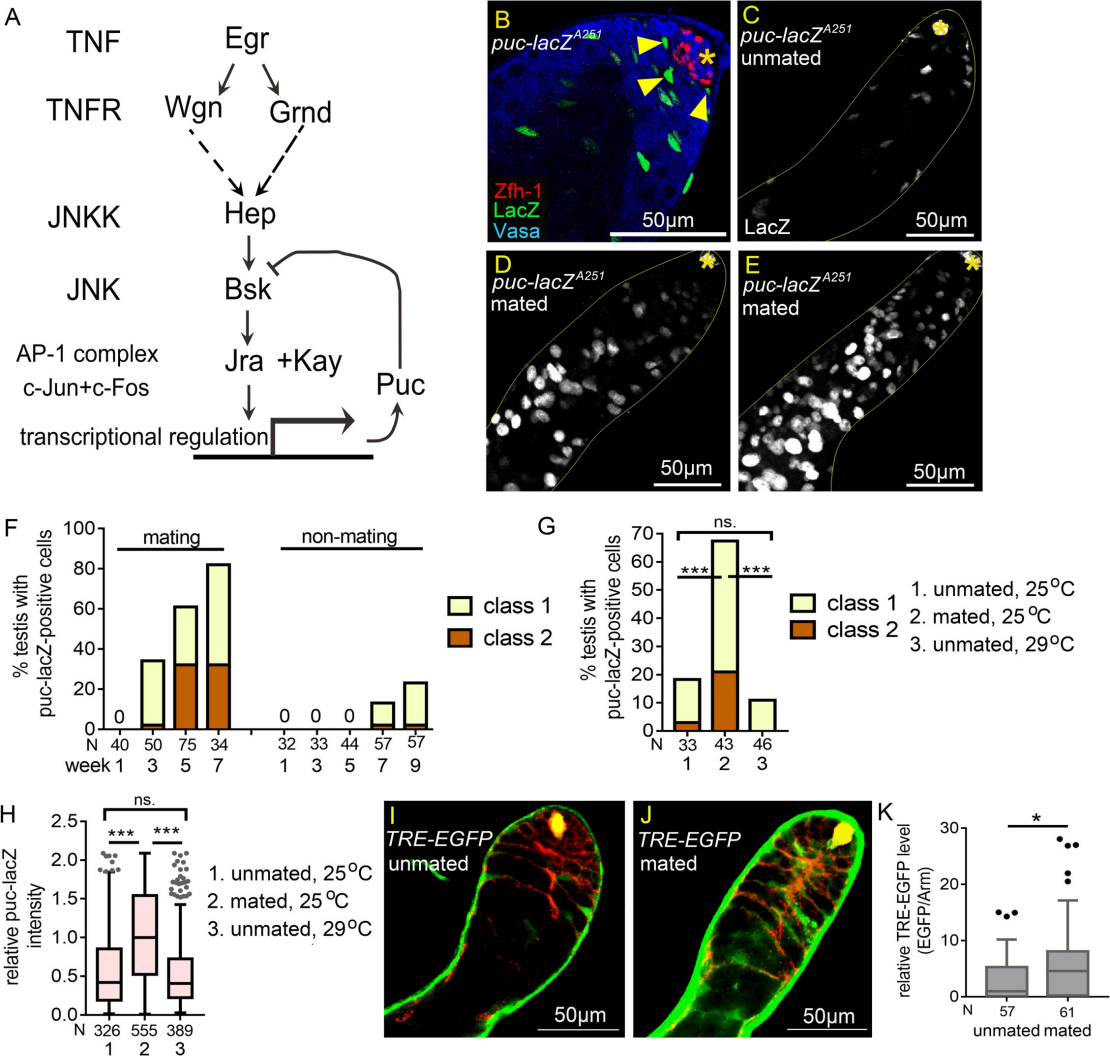
JNK signaling is hyperactivated in somatic cyst cells in mated testes. (A) Diagram of JNK signaling pathway. (B) Testis from 3-day-old *puc-lacZ*^*A251*^ male immunostained for β-galatosidase (green), co-stained for Zfh-1 (red) and Vasa (blue). Arrowheads indicate the LacZ-positive early cyst cells. (C-E) Testes from 4w-old *puc-lacZ*^*A251*^ males immunostained for β-galactosidase, co-stained for FasIII (asterisks). Testes exhibiting class 1 and class 2 of extra puc-lacZ-positive cells are shown in D and E. (F) The percentages of testes with extra puc-lacZ-positive cells were much higher in mated males than unmated males. N: number of testes scored. (G) Percentages of 4w-old testes with extra puc-lacZ-positive cells. Overproduction of puc-lacZ*-*positive cells was observed in mated testes, but not in unmated testes at both 25°C and 29°C. N: number of testes scored. (H) Box-and-whisker plots showing the relative anti-β-galactosidase intensity in single early cyst cells in 4w-old *puc-lacZ* testes. N: number of the puc-lacZ-positive cells scored. (I-K) Testes of *TRE-EGFP* immunostained for GFP (green), co-stained for Arm (red). (I and J) Reduced TRE-EGFP expression in cyst cells of the unmated 4w-old males (I) compared to the mated 4w-old males (J). (K) The relative TRE-EGFP intensity in early cyst cells of unmated and mated 4w-old males. N: number of the cyst cells scored. P-values were calculated by Mann-Whitney test in H and K, and by Chi-squared test in G, and P. ns: p>0.05, *p<0.05, **p<0.01, and ***p<0.001. Asterisks indicate the hubs. Mass-mating was conducted for experiments shown in D-H, J, and K. Unmated of 30 males per vial was in C, F, G, H, I, and K.

We found that reproduction induces hyperactivation of JNK signaling in the somatic cyst cells of the testes from mated males. Examination of the JNK signaling reporter *puc-lacZ*^*A251*^ found that it is expressed at high-levels in early cyst cells (Fig 5A and arrowheads in Fig 5B) [48]. Levels of *puc-lacZ*^*A251*^ expression in cyst cells were markedly reduced by RNAi knockdown of JNK, encoded by *basket* (*bsk*), in cyst lineage (Fig 5A, S3A, S3B and S3F Fig), indicating that JNK signaling is normally activated in somatic cyst cells. *bsk*-sensitive expression in early cyst cells was also demonstrated by another *puc* reporter *puc-lacZ*^*B48*^ (S3C–S3E, and S3G Fig). JNK signaling activation in cyst cells was also found via the EGFP reporter of TRE (TPA response element), that are the binding sites for the JNK effector AP-1 dimer (Fig 5I) [49].

While the testes of unmated males had approximately 20 *puc-lacZ*-positive cyst cells (Fig 5C), the numbers of *puc-lacZ*-positive cells were increased (more than 50 cells, see Methods) in more than 60% of the testes of mated males aged 4 to 7 weeks (Fig 5D–5F and column 2 in 5G). We quantified anti-β-galactosidase staining intensity of single *puc-lacZ*-positive early cyst cells to analyze levels of JNK signaling activity. As shown in Fig 5H, staining intensities were markedly higher in cyst cells of the testes of 4w-old mated males compared to those of age-matched unmated males. Reproduction-mediated JNK signaling hyperactivation is not solely induced by accelerated aging, as *puc-lacZ* levels in the cyst cells of unmated males cultured at 29°C to hasten aging [50, 51] were much lower than those of mated males cultured at 25°C (Fig 5H), even though the adult survival rates were comparable (S3H Fig). Similarly, elevated *TRE-EGFP* signals were also observed in testicular cyst cells in mated males compared to the unmated males (Fig 5I–5K). Together, these results indicate that reproduction activates JNK signaling in the testicular cyst cells of aged males.

**Fig 6:**
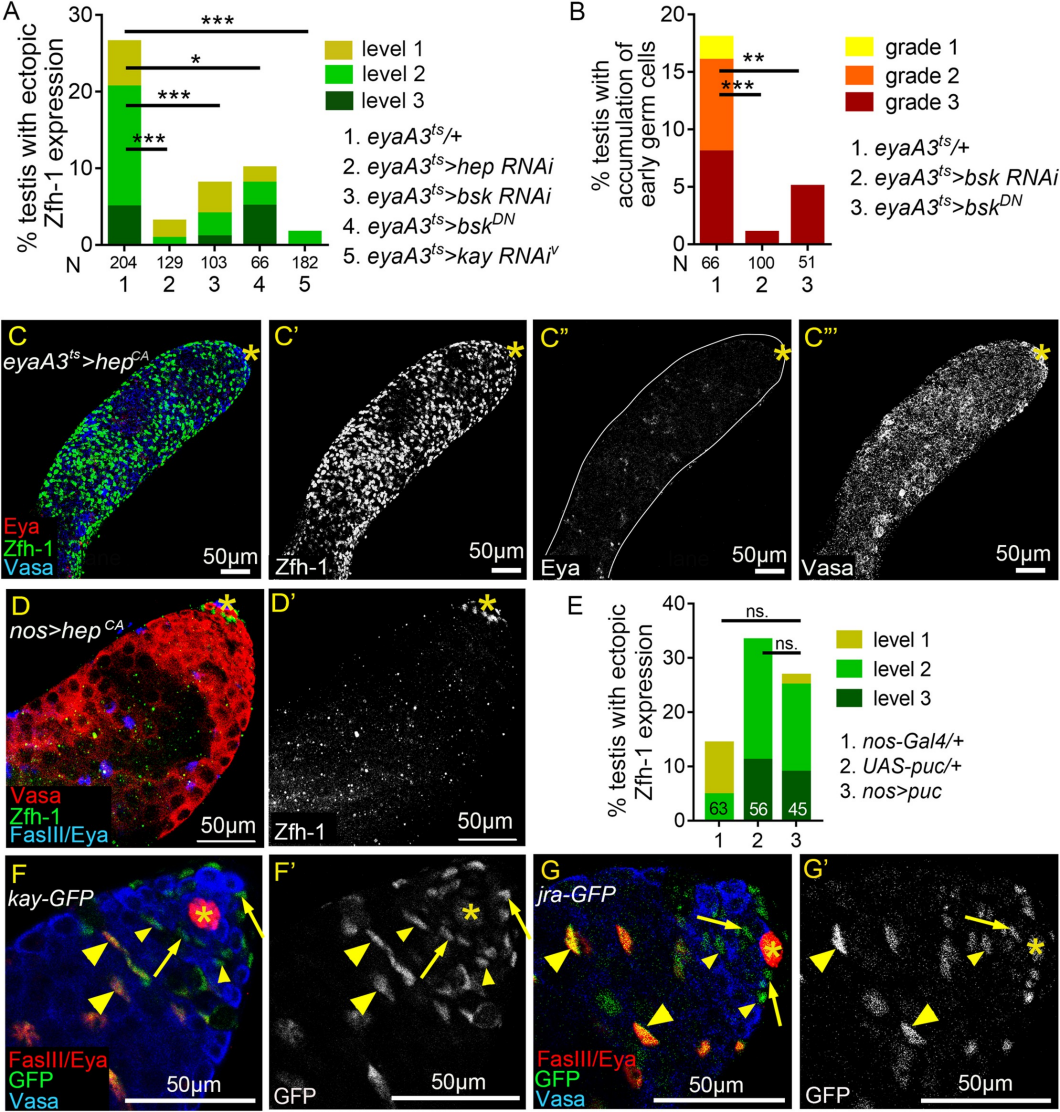
JNK signaling acts in cyst lineage to influence Zfh-1 expression. (A and B) Reducing JNK signaling activity in cyst cell lineage suppressed reproduction-induced ectopic Zfh-1 expression (A) and expansion of early germ cells (B). N: number of testes scored. Animals were maintained at 25°C during development. (C-C”‘) 1w-old testis immunostained for Zfh-1 (green in C and white in C’), co-stained with Eya (red in C, and white in C”) and Vasa (blue in C and white in C”‘). Constitutive activation of JNK signaling led to massive accumulation of Zfh-1-positive cells (C), loss of Eya expression (C”), and block of spermatocyte differentiation (C”‘). (D and D’) 1w-old testis immunostained for Zfh-1 (green in D; white in D’), co-stained for Vasa (red in D), and Eya and FasIII (blue in D). Zfh-1 expression and germ cell differentiation appeared normal upon overexpression of Hep^CA^ in germ cells. Animals were maintained at 25°C during development. (E) Reduction of JNK signaling in germ cells by Puc overexpression did not suppress ectopic Zfh-1 expression in testes of mated 4w-old males. N = number of the testes scored. (F-G’) 1-3-day-old testes immunostained for GFP (green in F and G, and white in F’ and G’), co-stained for Vasa (blue in F and G), and for Eya and FasIII (red in F and G). Both Kay-GFP and Jra-GFP proteins expressed from the BAC genomic clones were detected in the nucleus of cyst cells, but not in the nucleus of germ cells. Arrows mark the CySCs, and small and large arrowheads indicate early and late cyst cells, respectively. P-values in A, B, and E are obtained by Chi-squared test. ns: p>0.05, *p<0.05, **p<0.01, and ***p<0.001. Asterisks indicate the hubs. Mass-mating was conducted for experiments shown in A, B, and E.

Next, we investigated whether JNK signaling hyperactivation is the major cause of ectopic Zfh-1 expression and the early germ cell expansion induced by reproduction. RNAi-mediated silencing of JNK signaling components—including D-JNKK/Hemipterous (Hep), D-JNK/Basket (Bsk) and D-Fos/Kayak (Kay) (Fig 5A), in the cyst cell lineage via the *eyaA3*^*ts*^ system at 29°C markedly suppressed ectopic Zfh-1 expression in the testes of mated males, as also found for overexpression of dominant-negative Bsk (referred to as Bsk^DN^) (Fig 6A). Consistent with the role of cyst cells in controlling germ cell differentiation, expansion of undifferentiated germ cells was also suppressed by reducing *bsk* activity in cyst cells (Fig 6B). Conversely, forced expression of the constitutively active Hep (referred to as Hep^CA^) in the cyst cell lineage for seven days resulted in testes being filled with numerous ectopic Zfh-1-positive cells and small germ cells (Fig 6C–6C”‘). Only a few Eya-positive cells were present in these testes, indicating cyst differentiation was inhibited (Fig 6C”). Together, these results demonstrate that hyperactivation of JNK signaling in cyst cells is the major cause of the cyst cell and germ cell differentiation defects induced by reproduction.

It is shown by a recent paper that mating and starvation promotes spermatogonia de-differentiation to GSCs, and this requires JNK signaling activation in the germline [52]. Thus, we asked if JNK signaling activation in germ cells influences Zfh-1 expression. When JNK signaling was hyper-activated in germ cells by forced expression of Hep^CA^ for 7 days, neither ectopic Zfh-1 expression nor early germ cell expansion was observed (Fig 6D and 6D’). This may be due to the lack of downstream transcriptional factor AP-1 protein complex, Kay and Jra in the germ cell nucleus (Fig 5A and Fig 6F–6G’). In the testes of aged, mated males, block of JNK signaling activation in germ cells by mis-expression of Puc, a negative feedback regulator of JNK [53], did not significantly suppress ectopic Zfh-1 expression compared to the controls (Fig 6E). Together, these results show that JNK signaling activation in the germ cells does not influence Zfh-1 expression in both mated and unmated conditions.

### The testis sheath produces TNF Eiger

**Fig 7:**
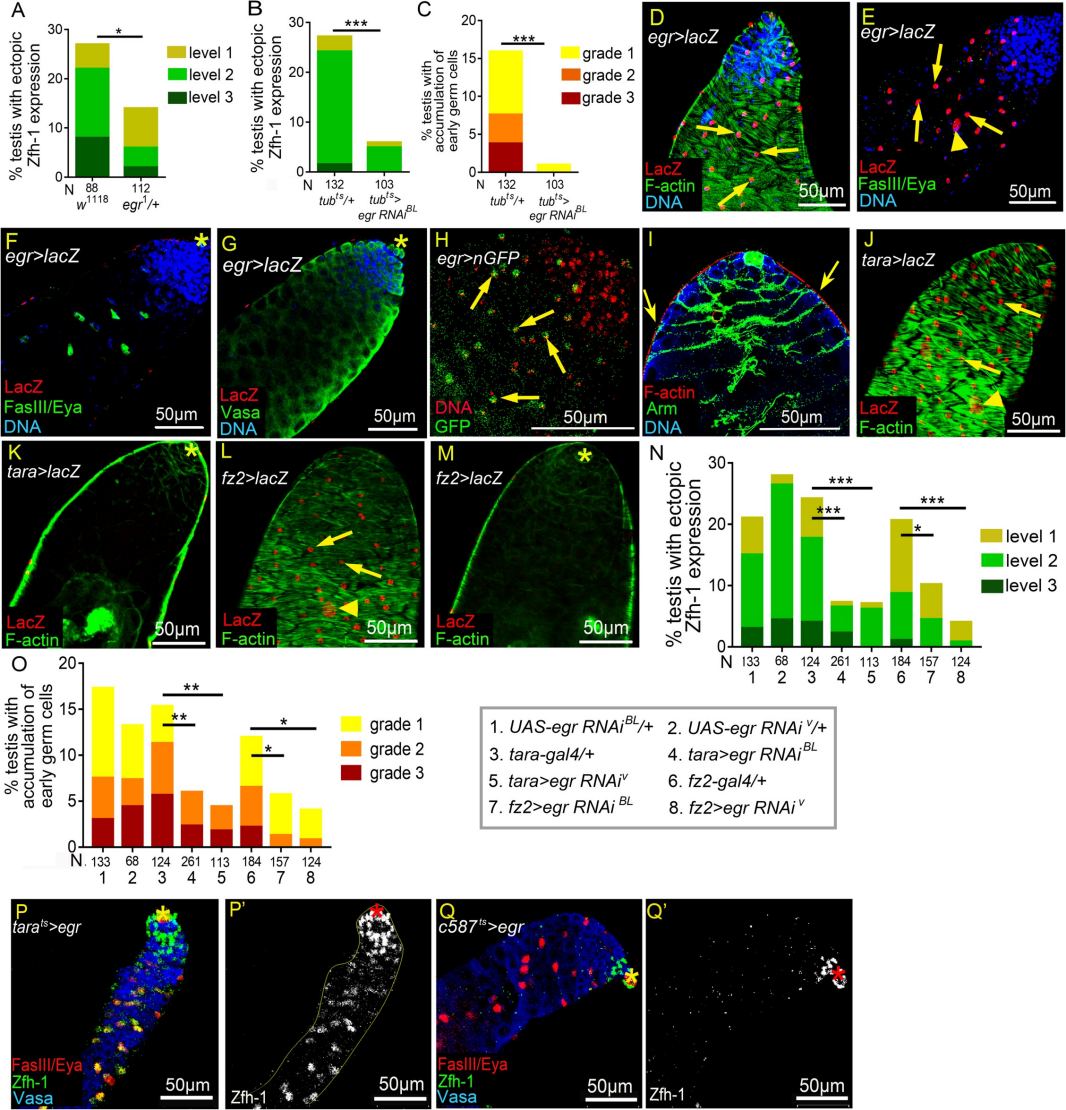
Egr induces ectopic Zfh-1 expression from testis sheath. (A-C) Percentages of testes with ectopic Zfh-1 expression (A and B) and accumulation of early germ cells (C) from mated 5w-old (A) and 4w-old (B and C) males. (A) One copy of *egr*^*1*^ allele significantly reduced the percentage of mated testes exhibiting ectopic Zfh-1 expression at 5w-old. N = number of the testes scored. (B-C) Suppression of reproduction-induced ectopic Zfh-1 expression (B) and accumulation of early germ cells (C) via ubiquitous knockdown of *egr*. (D-G) Testes from 1-3-day-old *egr-Gal4>UAS-lacZ* (*egr>lacZ*) males. (D) Testis immunostained for β-galactosidase (red), co-stained for F-actin (green) and DNA (blue). LacZ was expressed in testis muscle characterized by a chevron-like F-actin pattern. (E and F) An *egr>lacZ* testis immunostained for β-Galactosidase (red), co-stained for FasIII and Eya (green) and DNA (blue). (E) Thin optical section of the testis surface. LacZ was detected in the nuclei of muscles (arrows) and pigment cells (arrowhead). (F) Thin optical section of the testis interior. LacZ was not expressed in the cyst cells (Eya-positive cells, green). (G) LacZ was not expressed in the germ cells (Vasa-positive, green). (H) A testis from a 1-3-day old *egr>nGFP* male immunostained for DNA (red). GFP fluorescence was detected in the nuclei of testis muscles (arrows). (I) A testis from 1-3-day old *w*^*1118*^ male immunostained for F-actin (red), co-stained for Arm (green) and Vasa (blue). Cyst cell cytoplasm located right beneath the muscles (arrows). (J-M) Expression patterns of *tara-Gal4* (J and K) and *fz2-Gal4* (L and M). *UAS-LacZ* expression driven by the two Gal4 lines were activated in testicular muscles characterized with the Chevron-like F-actin patter (J and L). LacZ expression was absent in the interior of testes (K and M). (N and O) Suppression of ectopic Zfh-1 expression (N) and accumulation of early germ cells (O) by depletion of *egr* via *tara-Gal4* and *fz2-Gal4*. (P-O’) Testes from 14-day-old unmated males (30 males *vs*. 0 female) immunostained for Zfh-1 (green in P and Q; white in P’ and Q’), co-stained for FasIII and Eya (red in P and Q), and Vasa (blue in P and Q). Ectopic Zfh-1 expression in Eya-positive cyst cells was induced by *egr* overexpression via *tara*^*ts*^ driver (P-P’). Overexpression of *egr* in early cyst cells failed to trigger ectopic Zfh-1 expression (Q and Q’). Asterisks mark the hubs. P-values from Chi-squared test are shown in A-C, N, and O. *p<0.05, **p<0.01, and ***p<0.001. Mass-mating was conducted for results shown in A-C, N, and O.

To elucidate the mechanism by which reproduction activates JNK signaling in testes of aged males, we sought to identify the ligand that triggers this response. In *Drosophila*, JNK signaling can be activated by the TNF-type ligand Eiger (Egr) [54, 55] (Fig 5A). We were unable to examine the reproduction-induced phenotypes in null mutant *egr*^*1*^ testes due to the early lethality of *egr*^*1*^ mated males before week 4. However, the percentage of ectopic Zfh-1 expression was significantly decreased in the testes of heterozygous *egr*^*1*^*/+* 5w-old flies compared to control mated *w*^*1118*^ flies (Fig 7A). Depletion of *egr* in the whole body using the *UAS-egr RNAi*^*BL*^ line driven by the ubiquitously-expressing *tub-Gal4/Gal80*^*ts*^ (*tub*^*ts*^) eliminated *egr* expression in 1w-old adult flies (S4A Fig), and suppressed the ectopic Zfh-1 expression and the expansion of early germ cells in the testes from mated males (Fig 7B and 7C). Together, these results indicate that *egr* is essential for the reproduction-induced phenotypes we observed in the testes of male flies.

To investigate the source of Egr, we analyzed *egr* expression using the enhancer trap *egr-Gal4* line [56]. Strikingly, we found that *egr* was expressed in the outer layer of the testis, the testis sheath (Fig 7D and 7E). The testis sheath is composed of an outer layer of pigment cells and an inner layer of circular smooth muscle [20, 21]. We specifically detected nuclear β-galactosidase in the smooth muscles (arrows in Fig 7D and 7E) and pigment cells (arrowheads in Fig 7E) of testes from *egr>lacZ* males, that express LacZ via the *egr-Gal4* driver. By contrast, β-galactosidase was absent from their cyst cells and germ cells (Fig 7F and 7G). We detected the same expression pattern through direct detection of GFP fluorescence in *egr>nGFP* testes (arrows in Fig 7H). Smooth muscles enclose the testis, and the images obtained by optically thin sectioning showed that cyst cell cytoplasm lays right beneath the muscle cells (arrows in Fig 7I), indicating the close proximity of these two types of cells. To further confirm *egr* expression in the testis sheath, we depleted it using *tara-Gal4* and *fz2-Gal4*, both of which were specifically expressed in the smooth muscle and pigment cells in the testes (Fig 7J–7M) [57], and examined *egr* expression levels in whole testes by qRT-PCR. Knockdown of *egr* by either one of the two Gal4 lines via *UAS-egr RNAi*^*BL*^ markedly and significantly reduced the *egr* mRNA levels in testes (S4B and S4C Fig), together, indicating that *egr* is primarily expressed in the testis sheath.

Next, we tested the role of *egr* in reproduction-induced testis defects. Knockdown of *egr* in testis sheath by *tara-Gal4* or *fz2-Gal4* at 29°C via *UAS-egr RNAi*^*BL*^ and the previously characterized *UAS-egr RNAi*^*V*^ [58, 59] dramatically reduced the numbers of ectopic Zfh-1-positive cells and suppressed the accumulation of early germ cells in the testes of mated male flies (Fig 7N and 7O). When *egr* was overexpressed by *tara-Gal4* in unmated males, Zfh-1 expression was induced in Eya-positive cyst cells, a phenotype similar to what we observed in the testes of mated males exhibiting level-1 ectopic Zfh-1 expression (Fig 7P and 7P’, 2N, and column 3 in Fig 2M). In contrast, *egr* overexpression in the cyst cell lineage failed to induce ectopic Zfh-1 expression (Fig 7Q and 7Q’). Together, these results demonstrate that *egr* acts non-autonomously in the testis sheath to promote ectopic Zfh-1 expression in cyst cells.

**Fig 8:**
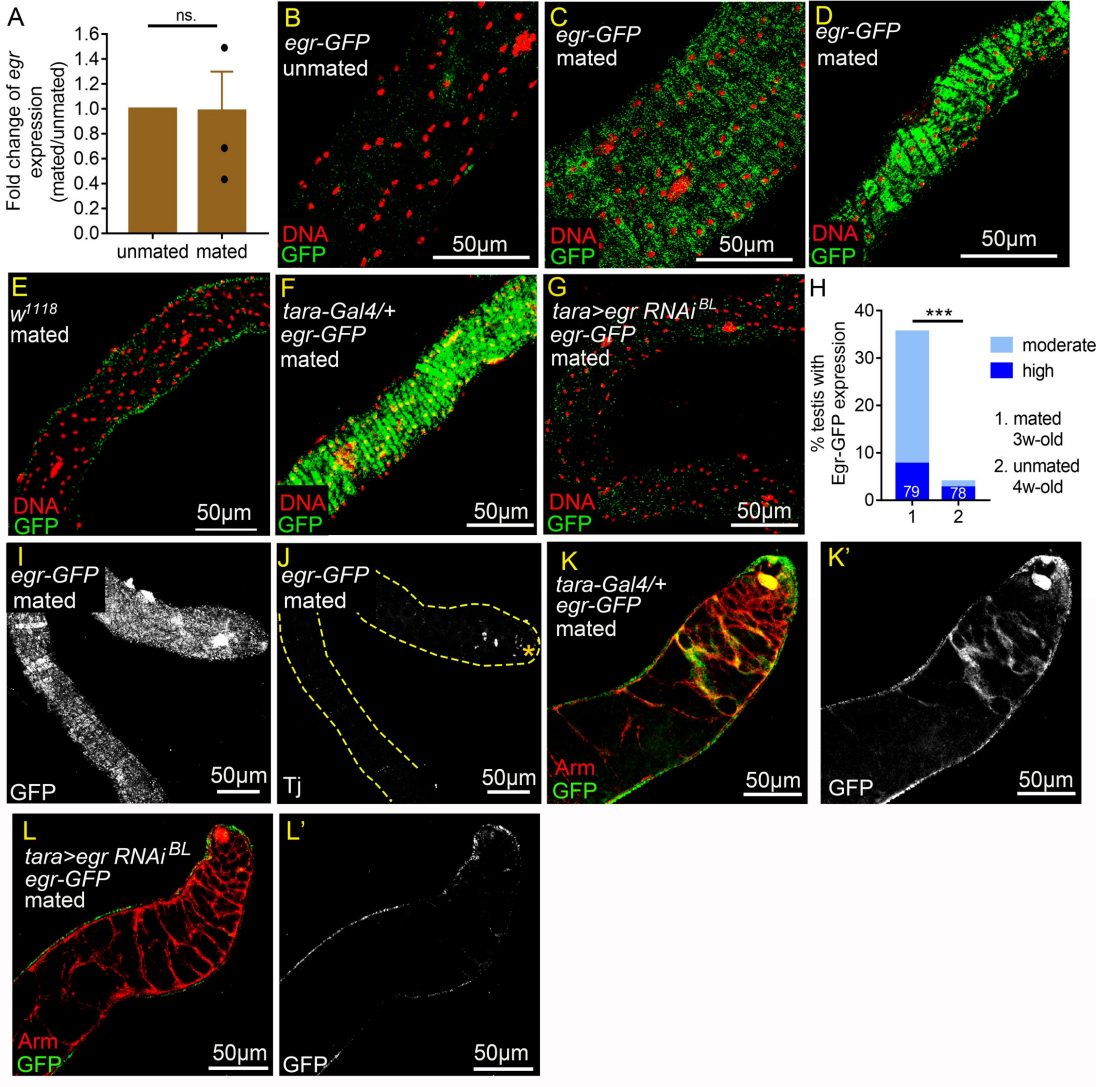
Reproduction triggers Egr accumulation in the testis smooth muscles. (A) qRT-PCR analysis of *egr* levels in testes from 3w-old males. Error bars represent SEM. N (The number of independent biological replicates) = 3. *egr* levels in testes were comparable between unmated and mated males. (B-G) Optical sections of the testicular muscle layer. All testes were immunostained for GFP (green), co-stained for DNA (red). (B-D) Testes from 3w-old *egr-GFP* males. (B) There were no specific anti-GFP signals detected in the muscles of unmated testes (30 *vs*. 0). (C and D) Egr-GFP was detected in moderate (C) or high (D) levels in the muscles of testes from males in the mass-mating scheme. (E) There were no specific anti-GFP signals in the testicular muscles of mated 3w-old *w*^*1118*^ males (10 *vs*. 20). (F and G) Testes from mated 4w-old males (10 *vs*. 20). Anti-GFP signals were completely abolished in *tara>egr RNAi* testes (G), while high levels of Egr-GFP was still observed in the *tara-Gal/+* control testis (F). (H) Percentages of testes with Egr-GFP accumulation in the muscles. Testes were from mated 3w-old males (1 *vs*. 6) or 4w-old single males kept in solitude (unmated, 1*vs*. 0). N = number of testes scored. (I and J) Confocal optical sections of a single testis from mated 3w-old *egr-GFP* male (1 *vs*. 6) immunostained for GFP, co-stained for Tj. Sections of testis surface and testis interior are shown in (I) and (J), respectively. While Egr-GFP accumulated in high levels in the muscles as early as week 3 (I), cyst cell differentiation was not yet disrupted, as shown by apically restricted Tj expression (J). Hub is marked by the asterisk. (K-L’) Testes from mated 3w-old *egr-GFP* males (10 *vs*. 20) immunostained with GFP (Green), co-stained with Arm (red). Egr-GFP was detected in some of the cyst cells (K and K’). Anti-GFP signals in cyst cells were abolished by depletion of *egr* in muscles by *tara-Gal4* (L and L’). P-value was calculated with paired *t* test in A and Chi-squared test for H. ns.: p>0.05 and ***p<0.001.

Although we found that decreasing and increasing *egr* levels in the testis sheath, respectively, suppressed and promoted ectopic Zfh-1 expression in cyst cells, *egr* mRNA levels, as measured by qRT-PCR, were comparable in testes of mated and unmated flies (Fig 8A), suggesting that the reproduction-induced mechanism operates downstream of *egr* mRNA production. We assessed a GFP-tagged Egr protein reporter expressed from a genomic fosmid clone [60] and notably found a reproduction-induced accumulation of Egr-GFP in the cytoplasm of testicular muscles of mated flies as young as 3w-old (Fig 8C and 8D). In some cases, high levels of Egr-GFP filled the muscle fibers throughout the whole testes (Fig 8D). By comparison, no specific Egr-GFP signals were detected in the testis muscles of 3w-old unmated Egr-GFP flies or 3w-old mated *w*^*1118*^ flies (Fig 8B and 8E). Furthermore, knockdown of *egr* by *tara-Gal4* in mated males abolished Egr-GFP signals in testicular muscles (Fig 8F and 8G), demonstrating the specificity of Egr-GFP signals in testicular muscles. Up-regulation of Egr-GFP in testis muscles was primarily induced by reproductive activity since it was observed in 35% (N = 79) of the testes of 3w-old males who mated with six females in single-male mating scheme (Fig 8H), whereas only 4% (N = 78) of testes from 4w-old males kept in solitude exhibited Egr-GFP up-regulation, despite having similar survival rates to the 3w-old mated males (Fig 8H and S5 Fig). Egr-GFP up-regulation in mated males is unlikely to be solely caused by accelerated aging because 90% of the flies were still surviving after mating for three weeks (S5 Fig). None of the testes of 3w-old mated males in which Egr-GFP had accumulated in the testicular muscle showed ectopic expression of Traffic jam (Tj), a CySC and early cyst cell marker (Fig 8I and 8J), suggesting that Egr accumulation in the testis muscle precedes induction of ectopic early cyst cells. These results indicate that reproduction can lead to Egr protein accumulation in testicular muscle before onset of the differentiation defect in cyst cells.

Although *egr* expression, shown by *egr-Gal4*, was specifically detected in the testicular smooth muscle (Fig 7D–7G), careful examination of Egr-GFP protein patterns found that GFP signals were also detected in some of the cyst cells (Fig 8K and 8K’). We speculate that the Egr-GFP in the cyst cells might be derived from muscles. When *egr* in testis muscle was knocked down by *tara-Gal4*, the Egr-GFP signals in the cyst cells in mated males were abolished (Fig 8L and 8L’), suggesting that the Egr protein detected on cyst cells is from testis muscles.

### Reproduction does not elevate Egr production in the fat body

In addition to testicular muscles, examination of *egr* expression found that it was also expressed in tissues that lie very close anatomically to testes; it was expressed in ISC/EB in the midgut [61] and in the fat body (S6A and S6A’ Fig). Knockdown of *egr* in ISC/EB by the *esg-Gal4*^*ts*^ system [62] did not suppress ectopic Zfh-1 expression in the testes from mated males (S6E Fig), suggesting that *egr* in intestine is not required for reproduction-induced Zfh-1 up-regulation. However, knockdown of *egr* in fat body by *lsp2-Gal4* [63, 64] mildly but significantly reduced the percentages of testes with ectopic Zfh-1 expression (S6E Fig). Examination of Egr-GFP found that Egr was expressed in very low levels in fat bodies in both mated and unmated males (S6B–S6C’ Fig), and *fz2-Gal4* was not expressed in the adult fat body (S6D and S6D’ Fig). In summary, these data indicate that *egr* in fat body is not essential for the reproduction-induced, *fz2-Gal4*-sensitive Zfh-1 up-regulation in the testes from mated males.

### TNFR Grnd in cyst cells is required for reproduction-associated phenotypes

**Fig 9:**
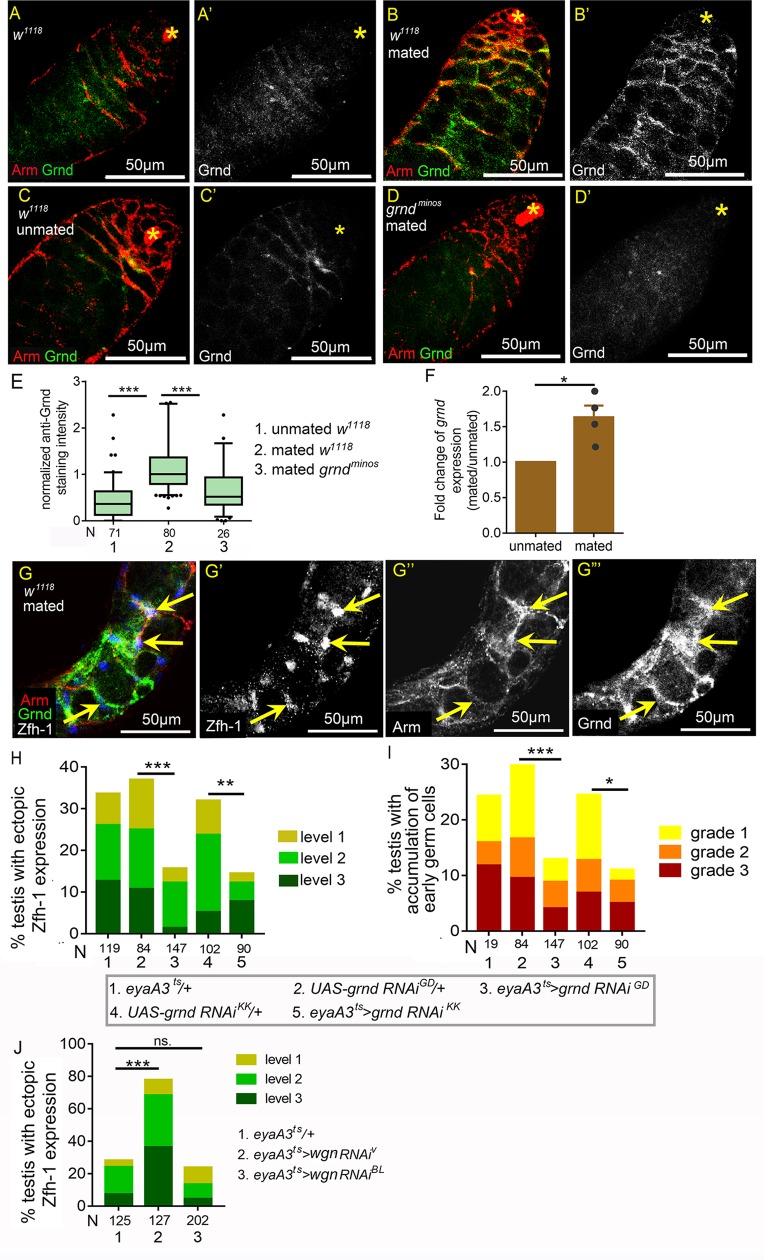
Expression of Grnd in cyst cells is elevated by reproduction. (A-D’) Testes immunostained for Grnd (green in A-D; white in A’-D’), co-stained for Arm (red in A-D). Asterisks mark the hubs. While there were almost no specific anti-Grnd signals in the testes from young males (1-3-day old) (A) and from 3w-old unmated males (C), Grnd was clearly detected in the cyst cell cytoplasm in testes of mated 3w-old males (B). Anti-Grnd signal was absent from the cyst cells in mated 3w-old *grnd*^*minos*^ testis (D). (E) Box-and-whisker plots showing normalized anti-Grnd/anti-Arm intensity in cyst cells. All the relative anti-Grnd intensities were further normalized to the median value for the testes of 3w-old mated males. (F) Fold changes of *grnd* transcript levels in testes from mated and unmated 3w-old males. Transcript levels were measured by qRT-PCR. The mean *grnd/rp49* levels in different samples were normalized to that in the unmated testes. N (biological repeat) = 4. Error bars represent SEM. (G-G”‘) A testis with level-1 ectopic Zfh-1 phenotype from mated 5w-old *w*^*1118*^ male immunostained for Grnd (green in G, and white in G”‘), co-stained for Zfh-1 (blue in G; white in G’) and Arm (red in G; white in G”). Arrows indicate the anti-Grnd signals in the ectopic Zfh-1-positive cells. (H and I) Suppression of reproduction-induced ectopic Zfh-1 expression (H) and expansion of early germ cells (I) in testes of mated 4w-old males by two independent *grnd RNAi* lines expressed via *eyaA3*^*ts*^ at 29°C. (J) Knockdown of *wgn* in cyst cells failed to reduce ectopic Zfh-1 expression in the testes of mated males. Statistic significances were calculated with Mann-Whitney test (E), paired *t* test (F), and Chi-squared test (H-I). *p<0.05, **p<0.01, and ***p<0.001. Mass-mating scheme were conducted for all experiments.

The proximity between the testis sheath and the cyst cells prompted us to ask whether Egr acts directly on the somatic cyst cells. To answer this question, we first examined expression of the TNF receptor Grnd by immunostaining [65]. In 1- to 3-day old testes, there was almost no anti-Grnd staining (Fig 9A and 9A’). Interestingly, Grnd was clearly detected in the cytoplasm of cyst cells in the testes of 3w-old mated flies, but not in age-matched unmated males (Fig 9B and 9C’). Specific anti-Grnd staining in cyst cells was not observed in the testes of 3w-old mated *grnd*^*minos*^ protein-null mutant flies, indicating the specificity of the staining pattern (Fig 9D and 9D’). Quantification of anti-Grnd staining intensity in the cyst cell cytoplasm revealed that Grnd levels were significantly higher in the testes of mated males compared to those from unmated flies (Fig 9E). Grnd could also be detected in the extra Zfh-1 cells in the testes with level 1 ectopic Zfh-1 expression (Fig 9G and 9G’”). Further examination of *grnd* transcript levels by qRT-PCR also confirmed up-regulation of *grnd* in the testes of mated male flies (Fig 9F).

We then assessed whether Grnd is involved in the manifestation of the testis phenotypes induced by reproduction. Silencing of *grnd* in cyst cells by two independent RNAi lines [65] substantially reduced the numbers of ectopic Zfh-1-positive cells and suppressed the expansion of early germ cells in the testes of mated flies (Fig 9H and 9I). In contrast, depletion of another *Drosophila* TNFR, Wengen (Wgn) [66] (Fig 5A), did not rescue the testicular phenotypes induced by reproduction (Fig 9J). Ectopic Zfh-1 expression was even enhanced in one of the *wgn* RNAi lines. Thus, reproduction induces ectopic Zfh-1 expression to block spermatogenesis in aged males via Grnd activation in cyst cells.

### JNK signaling is important for CySC maintenance in the testicular niche

**Fig 10:**
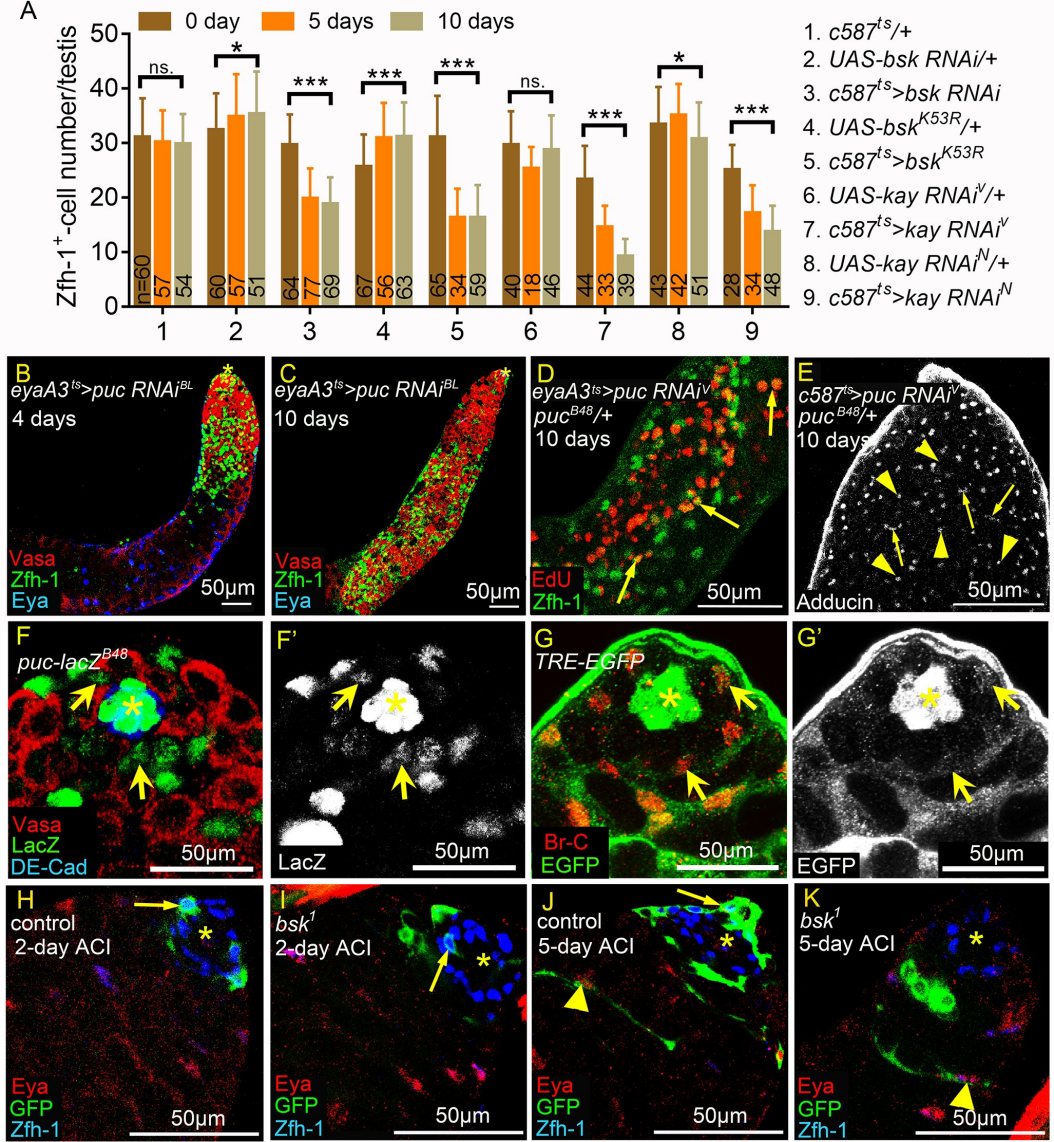
JNK signaling is required to maintain CySCs and early cyst cells. (A) Averages of Zfh-1-postive cell numbers per testis from unmated males at 0-, 5-, and 10-day-old. Numbers of testes scored are shown at the bottom of each column. Error bars represente SD. (B-E) *puc* knockdown testes. Animals were maintained at 25°C during development. (B and C) Testes immunostained for Zfh-1 (green), co-stained for Vasa (red) and Eya (blue). Testes were from males shifted from 25°C to 29°C for 4 days (B) or 10 days (C). 10-day incubation at 29°C led to excessive accumulation of Zfh-1-positive cells and small germ cells (C). (D) Testis labeled for EdU (red) incorporation and co-stained for Zfh-1 (green). Arrows mark Zfh-1 and EdU double-positive cells away from the hub, suggesting S phase progression in ectopic Zfh-1-positive cells. (E) Testis immunostained for Adducin to label spectrosomes (arrowheads) and thin fusomes (arrows). Numerous spectrosome-positive GSC/Gb-like cells and thin fusome-positive spermatogonia were observed in *puc* knockdown testes. (F-G’) Testes from 1w-old *puc-lacZ*^*B48*^ (F and F’) and *TRE-EGFP* (G and G’) males. (F and F’) Testis immunostained for β-Galactosidase (green in F and white in F’), co-stained for DE-cad (blue) and Vasa (red). LacZ was detected in nucleus of CySCs (arrows). (G and G’) Testis immunostained for GFP (green in G and white in G’), co-stained for Br-C (red) to mark CySCs and early cyst cells. GFP was detected in nucleus and cytoplasm of CySCs (arrows). (H-K) Testes with control or *bsk*^*1*^ clones marked by MARCM system. Asterisks indicate the hubs. Arrows indicate the GFP-positive CySC clones. Arrowheads mark the cyst cells with elongated cytoplasmic GFP. Scale bars = 50μm. (H and I) When dissected 2 days after clone induction (ACI), CySC clones (arrows) were observed in both *FRT*^*40A*^ control (H) and *FRT*^*40A*^ *bsk*^*1*^/*+* (I) testes. (J and K) When dissected 5 days ACI, cyst cell clones with elongated cytoplasm (arrowheads) were observed in both *FRT*^*40A*^ control (J) and *FRT*^*40A*^ *bsk*^*1*^/*+* (K) testes. CySC clones were lost in some *FRT*^*40A*^ *bsk*^*1*^/*+* testes (K). P-values were calculated with student *t* test in A. ns: p>0.05, *p<0.05, and ***p<0.001.

Finally, we investigated the functions of JNK signaling and the upstream Egr-Grnd ligand receptor complex in regulation of self-renewal gene expression in cyst lineage in unmated males. JNK signaling activity in early cyst lineage was reduced by depletion of *bsk* or *kay* via *c587*^*ts*^, or by forced expression of Bsk^K53R^ that blocks endogenous *bsk* activity [52, 67]. Reduction of JNK signaling activity for both five and ten days significantly decreased the total numbers of Zfh-1-positive cells (Fig 10A). In contrast, knockdown of *puc* in the cyst cell lineage for four days resulted in marked increases in numbers of Zfh-1-positive cells and small germ cells in the apical region of the testes (Fig 10B). Prolonged *puc* knockdown for 10 days led to testes being completely filled with Zfh-1-positive cells and small germ cells (Fig 10C), thereby recapitulating the phenotypes observed under Hep^CA^ expression (Fig 6C). Depletion of *puc* also led to more somatic cells undergoing S phase (Fig 10D) and an accumulation of spectrosome-containing GSC or Gb-like cells (arrowheads in Fig 10E). Taken together, our results support that JNK signaling normally maintains self-renewal gene expression in cyst lineage, and is negatively regulated by Puc.

Although at a lower level compared to early cyst cells, we also noticed the mild but specific expression of *puc-lacZ*^*B48*^ and *TRE-EGFP* in CySCs (arrows in Fig 10F and 10G’), suggesting that JNK signaling was also activated in CySCs. Analysis of the loss-of-function *bsk*^*1*^ and *bsk*^*2*^ mutants by a GFP-labeled Mosaic Analysis with a Repressible Cell Marker (MARCM) approach revealed that autonomous JNK signaling is required for CySC maintenance in the testicular niche. CySC clones are identifiable as GFP-positive, Zfh-1-expressing cells located one cell diameter away from the hub [68]. We dissected testes of control *FRT*^*40A*^ and the *bsk* mutant *FRT*^*40A*^*bsk*^*1*^ and *FRT*^*40A*^*bsk*^*2*^ flies at 2, 4–5, 6–7 and 10 days after clone induction (ACI). In *FRT*^*40A*^ controls, percentages of testes exhibiting CySC clones were maintained over time, ranging from 67% (N = 21) at 2 days ACI to 63% (N = 19) at 10 days ACI (arrows in Fig 10H and 10J, and Table 1). However, the percentages of testes possessing CySC clones were dramatically reduced over time in *FRT*^*40A*^ *bsk*^*1*^ flies, declining from 79% (N = 14) at 2 days ACI to only 6% (N = 16) at 6–7 days ACI (Fig 10I and 10K and Table 1). We also observed loss of CySC clones for the *bsk*^*2*^ allele that encodes a truncated protein [69] (Table 1). In some *FRT*^*40A*^ *bsk*^*1*^/+ testes that lacked GFP-labeled CySCs, we noted differentiating GFP-positive cyst cells away from the hub (arrowhead in Fig 10K). Since CySCs are the only cells that undergo mitosis-mediated recombination in the cyst cell lineage [37], these findings indicate that *bsk* mutant CySCs produce progeny that differentiate.

**Table 1:** JNK is required for CySC maintenance in the testicular niche.

	2 days ACI	4–5 days ACI	6–7 days ACI	10 days ACI
	Percentages with CySC clones^a^			
*FRT*^*40A*^*control*	66% (21)	75% (45)	65% (43)	63% (19)
*FRT*^*40A*^*bsk*^*1*^	78% (14)	38% (18)	6% (16)	NA
*FRT*^*40A*^*bsk*^*2*^	60% (25)	NA	7% (27)	NA

^a^. Percentages of testes with CySC clones (testes with GFP-positive CySCs/total testes with GFP-positive somatic cells).

The numbers in the parentheses are the total numbers of testes with GFP-positive somatic cells scored.

**Fig 11:**
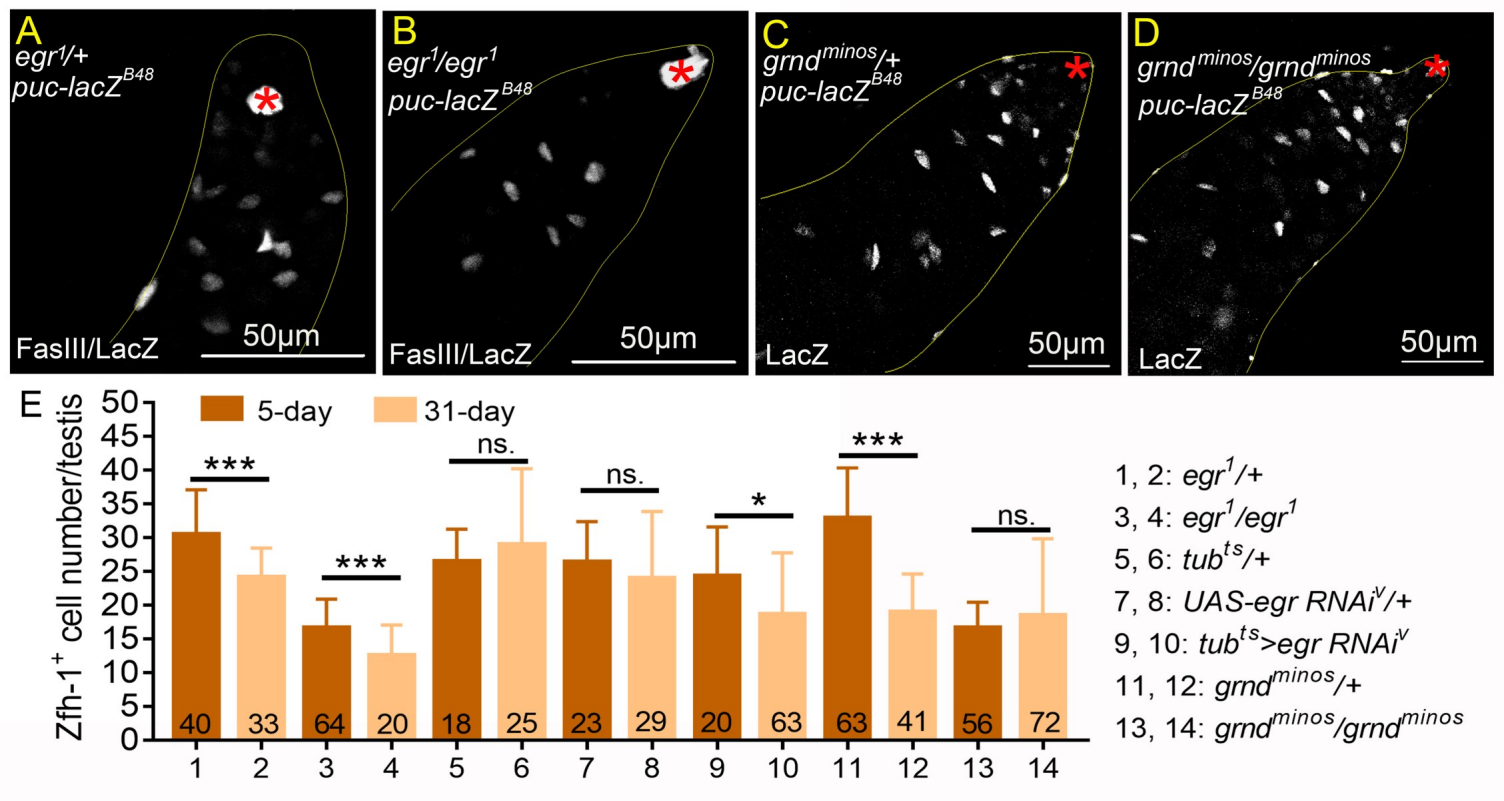
Egr-Grnd is not essential for endogenous JNK signaling activation in cyst cells. (A-D) Testes from unmated 1w-old *puc-lacZ*^*B48*^ males immunostained for β-Galactosidase, co-stained for FasIII to mark hubs (asterisks). LacZ expression was comparable between heterozygous controls and the homozygous mutants for *egr*^*1*^ (A and B) and *grnd*^*minos*^ (C and D). (E) Numbers of Zfh-1-positive cells in the testes from unmated 5- and 31-day-old males. Numbers of testes scored are shown at the bottom of each column. Statistic significances were calculated with student *t* test in E, and ns: p>0.05, *p<0.05, ***p<0.001. Unmated males (30 *vs*. 0) were examined in all experiments.

We then examined the roles of Egr and Grnd in testes in unmated males. Examination of *puc-lacZ* showed comparable LacZ expression in cyst cells between *egr*^*1*^ and heterozygous controls (Fig 11A and 11B). In testes from both *grnd*^*minos*^ null males and *grnd*^*minos*^*/+* males, *puc-lacZ* expression was also detected at similar levels (Fig 11C and 11D). These results together suggest that *egr* and *grnd* are dispensable for endogenous JNK signaling activity in cyst cells. In both heterozygous *egr*^*1*^*/+* and homozygous *egr*^*1*^ mutants, there was an approximately 20% reduction in the Zfh-1-positive cell numbers in the testes of 31-day-old flies compared to 5-day-old flies (Fig 11E). Ubiquitous knockdown of *egr* by the *tub*^*ts*^ system led to a mild decrease in Zfh-1 cell numbers (Fig 11E). Zfh-1-positive cell numbers were maintained in the testes of *grnd*^*minos*^ mutants (Fig 11E). To examine the role of *grnd* in CySC maintenance, *grnd* was depleted by expression of *grnd* RNAi in somatic clones. Unlike the dramatic reduction of CySC clones by lack of *bsk* within one week (Table 1), more than 60% of the CySC clone expressing *grnd RNAi* was maintained at 14-day ACI (Table 2). Together, our results suggest that *egr* and *grnd* are not essential to maintain CySCs and early cyst cells in the adult testes of unmated males.

**Table 2:** MARCM clones expressing *grnd* RNAi in the CySCs.

	3 days ACI	7 days ACI	14 days ACI
	Percentages with CySC clones^a^		
control	31.6% (152)	29.7% (195)	28.8% (184)
*UAS-grnd RNAi* ^*KK*^	36.2% (138)	34.1% (179)	21.8% (128)


^a^ Percentages of testes with CySC clones (testes with GFP-positive CySCs/total testes)

The numbers in the parentheses are the numbers of total testes scored.

## Discussion

**Fig 12:**
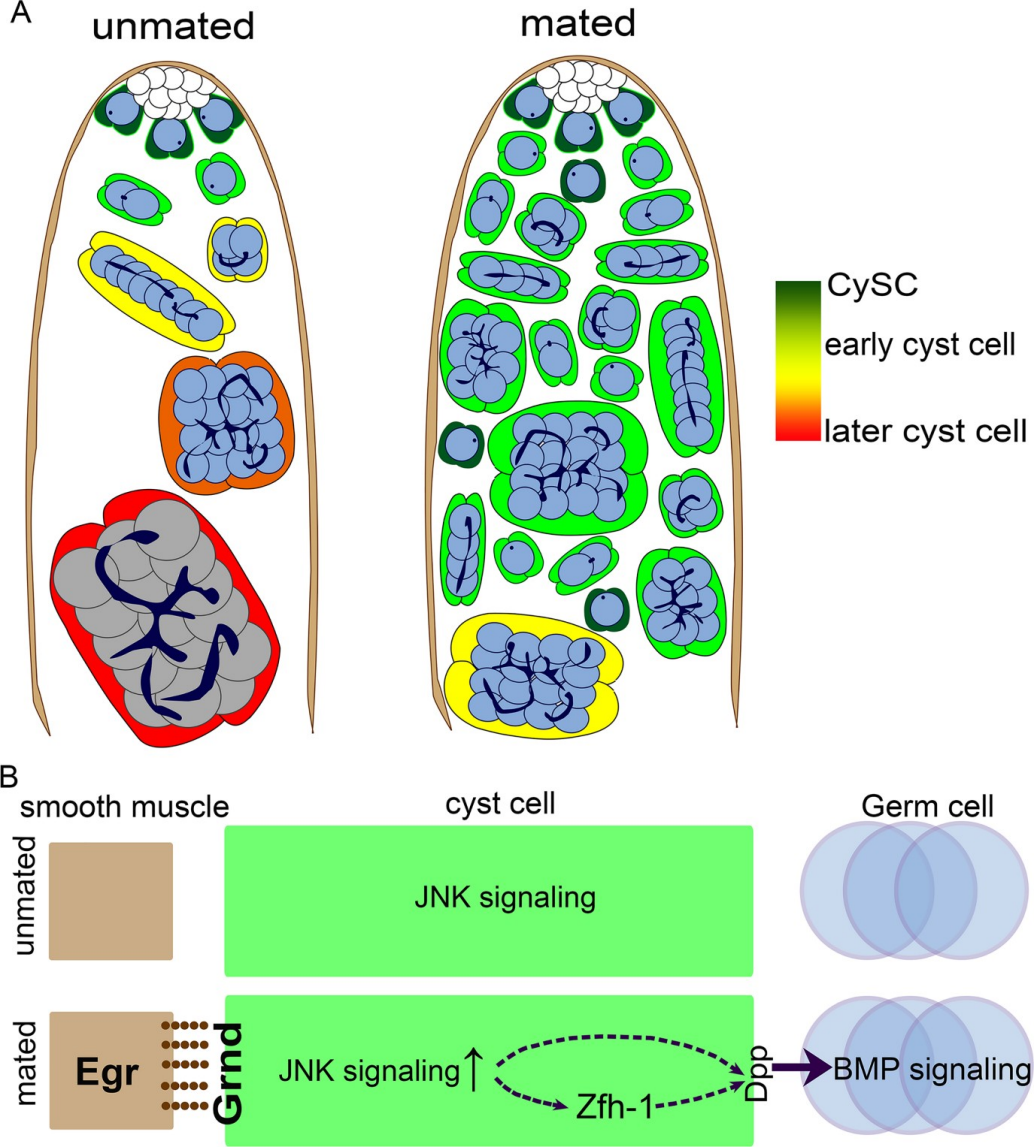
Summary and model of reproduction-induced accumulation of undifferentiated cells in the aged testes. (A) The schematic drawings of spermatogenesis in testes of unmated (left) and mated (right) aged males. Reproduction induces overproduction of CySC-like cells and early cyst cells due to extra cell divisions and blocked differentiation in aged males. Reproduction also leads to over-proliferation and accumulation of early germ cells at the expense of spermatocyte differentiation. (B) Models of reproduction-induced TNF-JNK signaling hyperactivation in regulation of spermatogenesis. In unmated testes (top), low-level JNK signaling is activated in cyst cells independent of Egr. In testes from mated males (bottom), Egr is accumulated abundantly in smooth muscles as early as 3w-old to act on induced TNFR Grnd. High-level JNK signaling activity induced by Egr up-regulates Zfh-1 expression and block differentiation in cyst lineage, that can be readily detected in testes from 5w-old males. Defect in germ cell differentiation is mediated by *dpp*-dependent BMP signaling activation.

Both males and females of diverse organisms incur costs such as reduced lifespan and future fecundity from increased reproduction [24, 25, 70–74]. Although recent studies have revealed molecular mechanisms underlying the reproductive tradeoff that modulates lifespan [75, 76], little is known about how reproduction influences spermatogenesis and stem cell homeostasis in the testes of aged animals. Our study demonstrates that reproduction can disrupt differentiation of stem cell progeny, leading to an accumulation of immature cyst cells and germ cells (right panel in Fig 12A). Strikingly, these reproduction-induced phenotypes result from Egr accumulation in testicular smooth muscle, which hyperactivates JNK signaling in the differentiating cyst cells, thereby up-regulating the self-renewal protein Zfh-1 and BMP signaling in CySC and GSC daughter cells, respectively (Fig 12B). Though maintenance of *Drosophila* CySCs and GSCs normally depends on the apically-localized testicular hub, our investigation reveals a novel role of testicular muscles as an induced signaling center to influence spermatogenesis and stem cell homeostasis in *Drosophila*.

### Combined effect of mating and aging in reproduction-induced phenotypes

Aging and reproduction influence each other. Reproduction reduces life span and accelerates aging through mechanisms such as compromising immune functions and decreasing protection from stress and damage [24, 75–77]. Aging reduces the numbers and activity of gonadal stem cells via both autonomous and non-autonomous mechanisms [35, 78–80]. Our studies show that reproduction blocks differentiation of early germ cell and early cyst cells in aged males but not in young males, and these phenotypes were rarely observed in very old (9w and 10w-old) males that never mated. Together, it reveals a combined effect of reproduction and aging in the reproduction-associated phenotypes, and adds a new layer of complexity in the interaction between aging and reproduction. Our study showed that the combined effect is at least on the level of Egr production in the testis muscle. Insulin signaling is critical to regulate reproduction and aging [81–84]. Interestingly, Egr is released from fat body to inhibit insulin production in the brain during nutrient shortage [59]. Potential communication between Egr and insulin signaling in the testis sheath and the role of insulin signaling in Egr production during reproduction needs further study.

Many studies have revealed cross talk between germ cells and somatic cells in the modulation of reproduction and aging, such as the regulation of germ cell division and differentiation by somatic cells, and modulation of organismal longevity, stress resistance, and trans-generational inheritance of survival advantages through germ cells [6, 38, 85–88]. We found that reproduction blocks future gamete production by signals from smooth muscles, revealing a somatic cell-mediated mechanism in negative feedback control of reproduction.

Our results reveal that mating has a detrimental effect in germline homeostasis in aged males. What is the evolutionary reasoning for this mechanism? Potentially, this mechanism could prevent production of large numbers of offspring form the same father, thus maintaining genetic diversity in the population. Furthermore, GSCs have to divide many times in order to generate substantial amounts of sperm for reproduction, and the likelihood of having a sperm with genetic mutations is much higher in aged fathers than the young fathers [89].

We also recorded a positive correlation between the levels of ectopic Zfh-1 expression and the numbers of available females in both single-male mating and mass-mating experiments. Since male flies fight for access to virgin females [90, 91], it is possible that the accumulated Zfh-1 cells seen in mass-mated males are largely due to male aggression. However, we found that the frequency of ectopic Zfh-1 expression in the *Canton-S* strain is quite comparable between mass-mating (10 males *vs*. 20 females) and single-male mating (1 male *vs*. 1–2 females) systems. In the *w*^*1118*^ strain, the frequency of Zfh-1-positive cell accumulation was higher in the mass-mating compared to the single-male mating system. Importantly, the frequency of ectopic Zfh-1 expression was much lower in a *w*^*1118*^ mass-mating system involving 25 males and only five females, which presumably induces more fighting and aggressive behavior among males. Moreover, Egr-GFP accumulation in testicular muscles was found in males subjected to either the single-male mating or mass-mating system. Thus, our results indicate that the up-regulation of TNF-JNK-Zfh-1 observed in mass-mating male flies is primarily induced by reproductive activity.

### JNK signaling activation in somatic cells and germline in *Drosophila* testis

Our study showed that reproduction hyperactivates JNK signaling in cyst lineage cells based on the following observations: (1) JNK signaling activity, assayed by reporters *puc-lacZ* and *TRE-EGFP* was up-regulated in early cyst cells in response to reproduction. (2) Knockdown of JNK signaling activity in cyst lineage suppressed mating-induced ectopic Zfh-1 expression and accumulation of early germ cells. (3) Forced activation of JNK signaling led to overproduction of Zfh-1-positive cells and early germ cells at the expanse of mature cyst cells and spermatocytes. (4) Reduction of TNF receptor Grnd, an upstream activator of JNK signaling [65] in the cyst lineage rescued reproduction-induced phenotypes. A recent paper showed that reproduction and starvation triggers spermatogonia de-differentiation to GSCs, a process which requires autonomous JNK signaling activation in germ cells [52]. While the JNK signaling activity in the cyst lineage can be easily observed by the JNK pathway reporters, the germline JNK signaling activation by reproduction and starvation can only be observed by lineage tracing via *puc-Gal4*. Thus, it appears that JNK signaling activation is normally maintained in the cyst lineage, and its activation in the germline is transient. We found that blockage or hyperactivation of JNK signaling in germ cells did not influence native nor ectopic Zfh-1 expression. In contrast, activating JNK signaling in cyst cells blocked germ cell differentiation. Our study also revealed that reproduction elevated TNFR Grnd expression in cyst cells. No detectable Grnd expression was observed in germ cells in mated males, suggesting that reproduction might activate somatic and germline JNK signaling in the testes via distinct mechanisms.

The cellular functions of JNK signaling are highly diverse [92–95]. During protein starvation, JNK signaling is upregulated in cyst cells to non-autonomously induce spermatogonia cell death [96]. Our data show that under normal nutritional conditions, hyperactivation of JNK signaling in cyst lineage cells by either mating or genetic manipulation leads to ectopic self-renewal gene expression and non-autonomous accumulation of early germ cells including GSC-like cells and spermatogonia. These results demonstrate that JNK signaling hyperactivation induces different cellular responses in cyst cells under different environmental conditions. Since JNK directly phosphorylates more than fifty different target proteins [97], it may activate a different set of downstream effectors in cyst cells in response to poor nutritional conditions [98, 99]. Differential cellular responses may also be dependent on the activities of additional components. For example, JNK hyperactivation induces apoptosis in *scribble*-deficient cells in imaginal discs. In *scribble* mutant cells that also express oncogenic Ras or Notch, JNK signaling is tumorigenic [100]. Therefore, protein starvation may activate additional signaling pathways or induce cellular changes in cyst cells, acting in concert with JNK signaling to give rise to non-autonomous spermatogonial cell death.

### TNF-JNK signaling modulates adult physiology

Though it is not essential for normal development, Egr plays important roles in maintaining *Drosophila* physiology and adult survival during damage or stress [58, 59, 64, 101, 102]. For example, *egr*^*1*^ mutant flies die much faster than control flies when challenged with several types of bacteria [102], and Egr is secreted from the fat body to inhibit insulin production during amino acid starvation. While Egr in the sheath blocked differentiation in the testes of mated males, our results suggest that Egr is required to decrease reproduction-induced death given that all mated *egr*^*1*^ mutants died before the age of four weeks. Thus, it is also likely that Egr is essential to resist reproduction-induced mortality in younger males, but comes at the expense of defective spermatogenesis in older mated males.

## Methods

### *Drosophila* stocks and fly husbandry

*w*^*1118*^, *Oregon R*, *Canton-S* and *yw* were used as wild-type, control strains. *egr*^*1*^ [54], *grnd*^*minos*^ [65] were null mutants. Gal4 lines used in this study are *c587-Gal4;tub-Gal80*^*ts*^ (referred as *c587*^*ts*^) [43], *eyaA3-Gal4;tub-Gal80*^*ts*^ (referred as *eyaA3*^*ts*^) [6], *tub-Gal4*, *tub-Gal80*^*ts*^ (referred as *tub*^*ts*^) (this study), *nos-Gal4* [103], *egr-Gal4* [56], *fz2-Gal4* (BL12796) [57], *tara-Gal4* (BL63905) [57]. *Dad-lacZ* [36], *puc-lacZ*^*B48*^ [53], *puc-lacZ*^*A251*^ (BL11173) [53], *TRE-EGFP* (BL59010) [49], *egr-GFP* (VDRC318615) [104], *jra-GFP* (BL50755), *kay-GFP* (BL38657) were reporter lines. *UAS-bsk*^*DN*^ (BL6409), *UAS-bsk*^*K53R*^ (BL9311) [52, 67], *UAS-egr* [54], *UAS-hep*^*CA*^ (BL9306), and *puc*^*EY09772*^ for *UAS-puc* (BL20627) [105] were UAS lines for overexpression.

The *UAS-RNAi* lines targeting *bsk* (VDRC34138) [106–108], *dpp* [BL25782 for *UAS-dpp RNAi*^*BL*^ [109] and NIG-FLY 9855R-1 for *UAS-dpp RNAi*^*N*^], *egr* [BL55276 for *UAS-egr RNAi*^*BL*^ and VDRC108814 for *UAS-egr RNAi*^*V*^ [58, 59]], *gbb* [NIG-FLY5562R-3 for *UAS-gbb RNAi*^*N*^ [110] and BL34898 for *UAS-gbb RNAi*^*BL*^], *grnd* [VDRC43454 and 104538 for *UAS-grnd RNAi*^*GD*^ and U*AS-grnd RNAi*^*KK*^, respectively [59, 65]], *hep* [NIG-FLY4353R-2 [111, 112]], *kay* [VDRC6212 for *UAS-kay RNAi*^*v*^ [113, 114] and NIG-FLY 15507R-2 for *UAS-kay RNAi*^*N*^ [114]], and *puc* (BL34392 for *UAS-puc RNAi*^*BL*^ and VDRC3018 for *UAS-puc RNAi*^*V*^) were all from Vienna *Drosophila* Resource Center (VDRC), Bloomington *Drosophila* Stock Center (BL), or the National Institute of Genetics-Fly Stocks (NIG-FLY). For all Gal4/RNAi experiments, female Gal4 flies were crossed with male RNAi lines. Female Gal4 flies and male UAS-RNAi flies were outcrossed to *w*^*1118*^ to serve as the control Gal4/+ and control UAS-RNAi/+ lines, respectively.

Adult flies were maintained in culture vials of 24 mm diameter and 50 mm height in an incubator with a 12h: 12h light: dark cycle at 25°C, unless otherwise indicated. For the mating experiments, flies were transferred to fresh vials twice per week. Females were replaced with virgin females every week. For the experiments expressing RNAi or transgenes with or without *tub-Gal80*^*ts*^, flies were maintained at 18°C-20 ^o^C to suppress Gal4 expression during development, unless otherwise noted. Adult flies were then moved to 25°C or 29°C for Gal4-mediated expression. For depletion of *dpp*, *gbb*, *hep*, *bsk*, or *kay* by *eyaA3*^*ts*^ and *nos-Gal4*, 3w-old adult flies were incubated at 29°C for 10 days before dissection. To silence *egr* by *tub*^*ts*^, *tara-Gal4*, *fz2-Gal4*, and to silence *grnd* by *eyaA3*^*ts*^, 2w-old adult flies were incubated at 29° C for 17 days before dissection.

### Antibodies and immunostaining

The following primary antibodies were used: mouse anti-Adducin (DHSB, 1:5), mouse anti-Arm (DHSB, 1:100), mouse anti-Bam (DSHB,1:10), mouse anti-β-Galactosidase (DHSB, 1:100), rabbit anti-β-Galactosidase (Cappel, 1:100), mouse anti-Eya (DHSB, 1:20), mouse anti-Fasciclin III (DSHB, 1:100), rabbit anti-PH3 (Millipore, 1:2000), guinea pig anti-Grnd (1:200)[65], rabbit anti-pMad (1:2000) [115], guinea pig anti-Tj (1:1000) [116], goat anti-Vasa (Santa Cruz dc-13, 1:250), rabbit anti-Zfh-1 (1:5000) [6], and rabbit anti-GFP (Invitrogen A11122, 1:250). Secondary antibodies conjugated to Alexa 488- (Molecular Probes), Cy3 or Cy5 (Jackson ImmunoResearch) were used at 1:250. Hoechst 33342 was used to stain DNA at 1:500 for 15 minutes. F-actin was stained with Alexa Fluor 568 Phalloidin (1:200) (Invitrogen) for 15 min before mounting.

Testes were dissected in 1xPBS, fixed in 4% paraformaldehyde and washed in 0.3% PBT. Before antibody staining, testes were permeabilized in 0.3% PBT containing 0.3% NaDOC for 30 minutes. Testes were incubated overnight at 4°C with primary antibodies diluted in 0.3% PBT containing 0.3% BSA, followed by three washes and incubation with secondary antibody for 2 hours at room temperature. For staining with anti-Grnd antibody, testes were blocked overnight in 0.3% PBT containing 0.3% BSA at 4°C, followed by overnight incubation with pre-absorbed anti-Grnd antibody (1:200) at 4°C. Pre-absorption was achieved by incubating overnight the anti-Grnd antibody with 50–70 pairs of adult *grnd*^*minos*^ testes in 50 μl 0.3% PBT containing 0.3% BSA at 4°C.

### Classification of reproduction-induced testes defects

Ectopic Zfh-1-positive cells were defined as cells localized at least 200 μm (four times the average distance from the testis tip to the basal end of the spermatogonia in the testes of 5w-old unmated males) away from the testis tip. Level 1 ectopic Zfh-1 expression was defined as testes having total numbers of Zfh-1-positive cells below 50 but exhibiting ectopic localization of Zfh-1 positive cells. Level 2 and 3 ectopic Zfh-1 expression were defined as total numbers of Zfh-1-positive cells being between 50–200 and more than 200, respectively.

The phenotype of accumulation of early germ cells was defined as instances where the distribution of early germ cells (small DNA-bright Vasa-positive cells) extended more than 200 μm from the testis tip to the basal end of the early germ cells. Phenotypic severity was categorized into grades 1, 2, and 3 according to distributions ranging between 200–400 μm (grade 1), 400–600 μm (grade 2), and more than 600 μm (grade 3). Increase of *puc-lacZ* cell numbers was classified as class 1 or 2 based on total numbers of *puc-lacZ* cells being between 50–200 and more than 200, respectively.

### Mosaic analysis

The MARCM system was used to create positively marked clones for *bsk*^*1*^, *bsk*^*2*^ and wild type control flies. We used flies of the following genotypes to generate clones:

*hsFLP, UAS-mCD8-GFP/Y;FRT^40A^bsk^1^/FRT^40A^tub-gal80;tub-gal4/+*,

*hsFLP, UAS-mCD8-GFP/Y;FRT^40A^bsk^2^/FRT^40A^tub-gal80;tub-gal4/+*,

*hsFLP*, *UAS-mCD8-GFP/Y;FRT*^*40A*^*/FRT*^*40A*^*tub-gal80;tub-gal4/+*.

Adult males of 1–3 days old were subjected to three heat shocks at 37°C for 30 minutes. After heat shocks, flies were kept at 25°C until dissection. The percentage of CySC clones at each time-point represents the number of testes with GFP-positive CySCs divided by the total number of testes with GFP-positive cyst lineage cells.

The random clone system was used to create positively marked clones for *grnd RNAi* and the control. We used flies of the following genotypes to generate clones:

*hsFLP, act<y+<Gal4*, *UAS-GFP/UAS-grnd RNAi^KK^*

*hsFLP*, *act<y+<Gal4, UAS-GFP/+*

Adult males of 1–3 days old were subjected to three heat shocks at 37°C for 30 minutes. After heat shocks, flies were kept at 25°C until dissection. The percentage of CySC clones at each time-point represents the number of testes with GFP-positive CySCs divided by the total number of testes scored.

### Quantitative RT-PCR

Total RNA was extracted from 70 to 100 pairs of adult testes (Fig 8A, Fig 9F, S4B Fig and S4C Fig) or from 20 to 25 adult flies (S4A Fig) using TRIzol RNA Isolation Reagents (Invitrogen). cDNAs were prepared using MMLV reverse transcriptase (Invitrogen). Quantitative PCR was performed using an iQ5 Gradient Real Time SYBR-Green PCR system (Bio-Rad). The amount of target RNA was normalized to the reference gene *rp49*. PCRs were performed using the following pairs of primers: *rp49*, 5’-ATCGG TTACG GATCG AACAA-3’ and 5’-GACAA TCTCC TTGCG CTTCT-3’ [117]. *egr*, 5’-TCGTC GATAA TCTCC AGCAG-3’ and 5’-GGATA GTCGA TGAGG GCATT-3’; and *grnd*, 5’-ACTAC GATGC CTTTC TGTGC-3’ and 5’-AGGAT CAGCT GCTGG GTATT-3’. Quantitations were performed in triplicates.

### EdU labeling

Testes were dissected in Schneider’s *Drosophila* medium (Invitrogen), followed by incubation in Schneider’s medium containing 100 μM EdU (Invitrogen) for 30 minutes at room temperature. Testes were then fixed in 4% formaldehyde for 15 minutes and stained with primary antibodies as described above. To visualize EdU, testes were incubated with Alexa 555-azide for 30 minutes at room temperature.

### Quantification of fluorescence intensity

To quantify fluorescence intensities, the testes were processed in parallel for the entire set, and the confocal images were obtained with the same confocal settings. To determine the fluorescence intensity, we selected the areas within single confocal sections using the Freehand tool in Image J software.

To quantify the anti-β-galactosidase fluorescence intensity of *puc-lacZ*, we considered the mean intensity of each cyst cell as being the mean intensity of a 25–35 μm^2^ nuclear area after subtracting the background intensity. We measured 8–30 *puc-lacZ*-positive cells in each testis. All anti-β-galactosidase intensities were normalized to the median value for the testes of 4w-old mated males.

To quantify fluorescence intensities of anti-GFP (TRE-EGFP), anti-Grnd and anti-Arm staining, we considered the mean fluorescence intensity of each cyst cell as being the mean intensity of a 30–40 μm^2^ cytoplasmic area. The same areas were used to measure anti-GFP/anti-Arm or anti-Grnd/anti-Arm intensities. On average, we measured 6 to 8 cyst cells next to 8- to 16-cell spermatogonia clusters in each testis. The relative anti-Grnd intensity of each cyst cell represents the mean anti-Grnd intensity (after subtraction of the background anti-Grnd intensity) divided by the mean anti-Arm intensity (after subtraction of the background anti-Arm intensity). The same normalization method was also applied to the analysis of anti-GFP intensity in cyst cells.

### Statistical analysis

P values were generated using Chi-squared test to compare ectopic Zfh-1 expression, accumulation of early germ cells, extra puc-lacZ-positive cells, survival rate, and Egr-GFP expression between treatments. Statistically significant differences in immunofluorescence intensities, including for anti-GFP, anti-Grnd staining, and anti-β-galactosidase staining, were calculated by Mann-Whitney nonparametric test. Results for Zfh-1-positive cell number were calculated by student *t* test in Figs 10A and 11E. Results from qRT-PCR were statistically assessed by student *t* test in S4A and S4B Fig, and paired *t* test in Fig 8A, Fig 9F and S4C Fig. For all statistical analyses, ns: not significant (p>0.05), *p<0.05, **p<0.01, and ***p<0.001.

## Supporting information

S1 Table Numerical data for graphs and summary statistics. (XLSX)

S1 Fig Reproduction reduces lifespan in male flies. Survival rate of *w*^*1118*^ males during aging. Survival rate was markedly reduced in single males mated with 6 virgin females (1 *vs*. 6) compared to males kept in solitude. Numbers of the flies scored are shown in parentheses.
(TIF)

S2 Fig Comparable reproductive activity among males of *w^1118^*, *Canton-S* and *Oregon R*. In the fertility assay, one male and six virgin females were kept together in the vial for 24 hours, and the numbers of females laying fertilized eggs were examined. In all three genotypes, more than 70% males mated successfully with all six females within 24 hours.
(TIF)

S3 Fig JNK signaling components are specifically expressed in somatic cells. (A, B, D, E) Testes from 1w-old *puc-lacZ*^*A251*^ (A and B) and *puc-lacZ*^*B48*^ (D and E) males immunostained for β-Galactosidase. LacZ signals were markedly reduced by knockdown of *bsk* in cyst cells for 7 days. Animals were maintained at 25°C during development. (C) A testis from 3-day-old *puc-lacZ*^*B48*^ male immunostained for β-Galactosidase (green), co-stained for Zfh-1 (red) and Vasa (blue). Asterisks mark the hubs. (F and G) Box-and-whisker plots showing normalized LacZ intensity in cyst cells. N: number of the cells scored. Depletion of *bsk* significantly decreased the LacZ expression intensity in cyst cells. Animals were maintained at 25°C during development. (H) Survival rate of 4w-old *puc-lacZ*^*A251*^ males. The unmated 4w-old males a 29°C showed comparable survival rate as mated 4w-old males at 25°C. Numbers of males scored are shown at the bottom of each column. P-values were calculated with Mann-Whitney test in F and G, and Chi-squared test in H. ns: p>0.05, *p<0.05, **p<0.01, and ***p<0.001. Mass-mating (10 *vs*. 20) and unmated (30 *vs*. 0) were conducted for H.
(TIF)

S4 Fig *egr* transcripts are primarily detected in testicular muscles. (A) qRT-PCR analysis of *egr* levels in the 1w-old flies. Error bars represent SD. (B and C) qRT-PCR analysis of *egr* transcript levels in testes from 1w-old males. The means of *egr/rp49* levels from different samples were normalized to that of *UAS-egr RNAi* testes. Marked decrease of *egr* mRNA levels was observed in the testes of *egr* knockdown by *fz2-Gal4* (B) and *tara-Gal4* (C). (C) N (number of independent biological replicates) = 3. Error bars represent SEM. *P* values shown in A and B are obtained from student *t* test. P-value shown in C is obtained from paired *t* test. *p<0.05, **p<0.01, and ***p<0.001.
(TIF)

S5 Fig Survival rate of *egr-GFP* flies during aging. The survival rate of the unmated (1 *vs*. 0) *egr-GFP* males at 4w-old was comparable to that of mated 3w-old males (1 *vs*. 6). The numbers of males are shown in parenthesis.
(TIF)

S6 Fig Egr expression in adult fat body. (A and A’) Fat body from 1w-old *egr-Gal4>UAS-nGFP* male immunostained for GFP (green in A; white in A’), co-stained for Arm (blue) and DNA (red). *egr* was expressed in adult fat body. (B-C’) Fat bodies from 3w-old *egr-GFP* males immunostained for GFP (green in B and C; white in B’ and C’), co-stained for DNA (red in B and C) and Arm (blue in B and C). Comparable, low-level GFP expression was observed in fat bodies from unmated (B and B’) and mated (C and C’) males. (D) Fat body from 1w-old *fz2-Gal4>UAS-nGFP* male immunostained for GFP (green in D and white in D’), co-stained for DNA (red in D). No nuclear GFP signals were detected in fat body. (E) Percentages of testes with ectopic Zfh-1 expression from mated 4w-old males. No or mild suppression was observed by *egr* knockdown, respectively, in ISC/EB via *esg*^*ts*^ and in fat body via *lsp2-Gal4*. P values were calculated with Chi-squared test. ns: p>0.05, *p<0.05, **p<0.01, and ***p<0.001. Mass-mating was conducted in this experiment.
(TIF)
