# Supplementary figures and images for: Reproduction disrupts stem cell homeostasis in testes of aged male Drosophila via an induced microenvironment
Source: PLoS Genet. 2019 Jul 11;15(7):e1008062. doi: 10.1371/journal.pgen.1008062 (PMC6622487; doi:10.1371/journal.pgen.1008062)

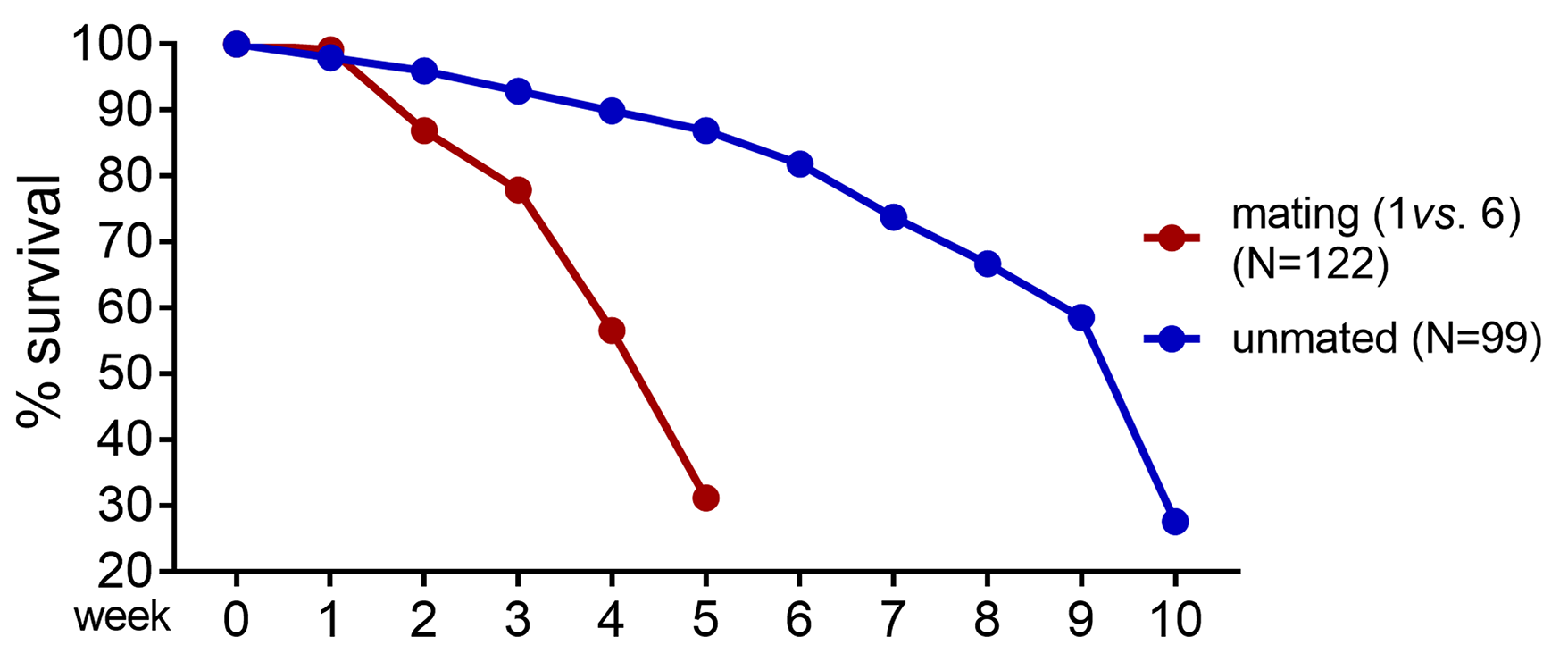

Supplement: S1 Fig — Survival rate of w1118 males during aging. Survival rate was markedly reduced in single males mated with 6 virgin females (1 vs. 6) compared to males kept in solitude. Numbers of the flies scored are shown in parentheses. (TIF) [file pgen.1008062.s002.tif]

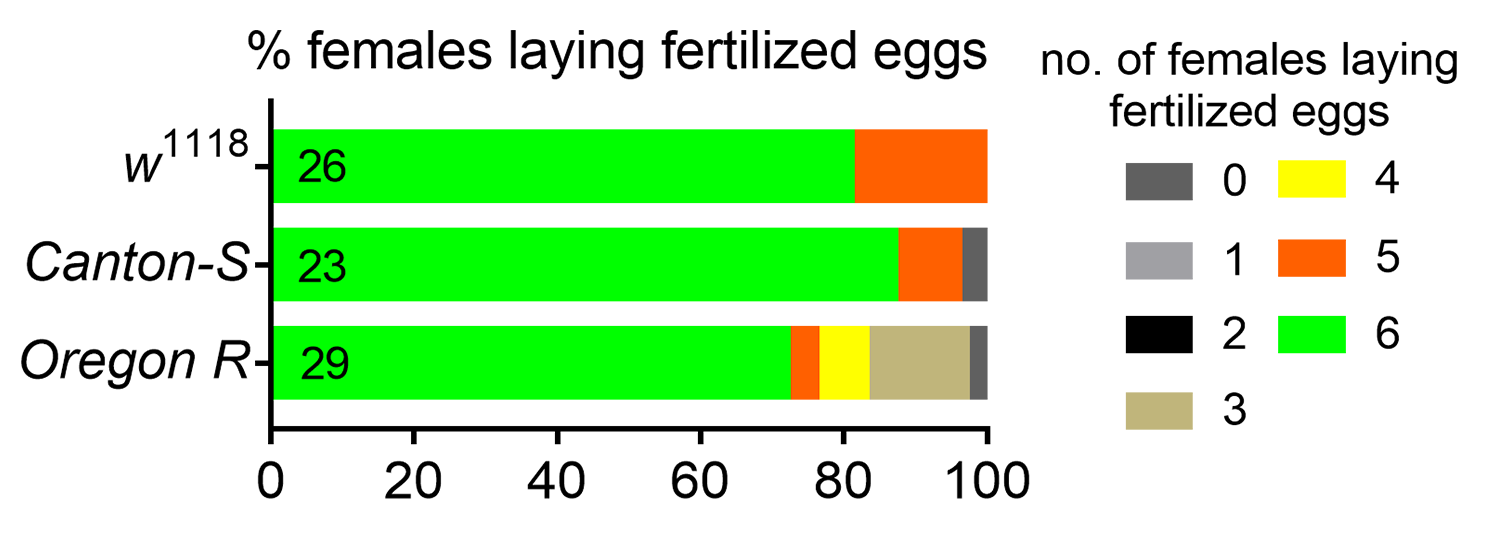

Supplement: S2 Fig — In the fertility assay, one male and six virgin females were kept together in the vial for 24 hours, and the numbers of females laying fertilized eggs were examined. In all three genotypes, more than 70% males mated successfully with all six females within 24 hours. (TIF) [file pgen.1008062.s003.tif]

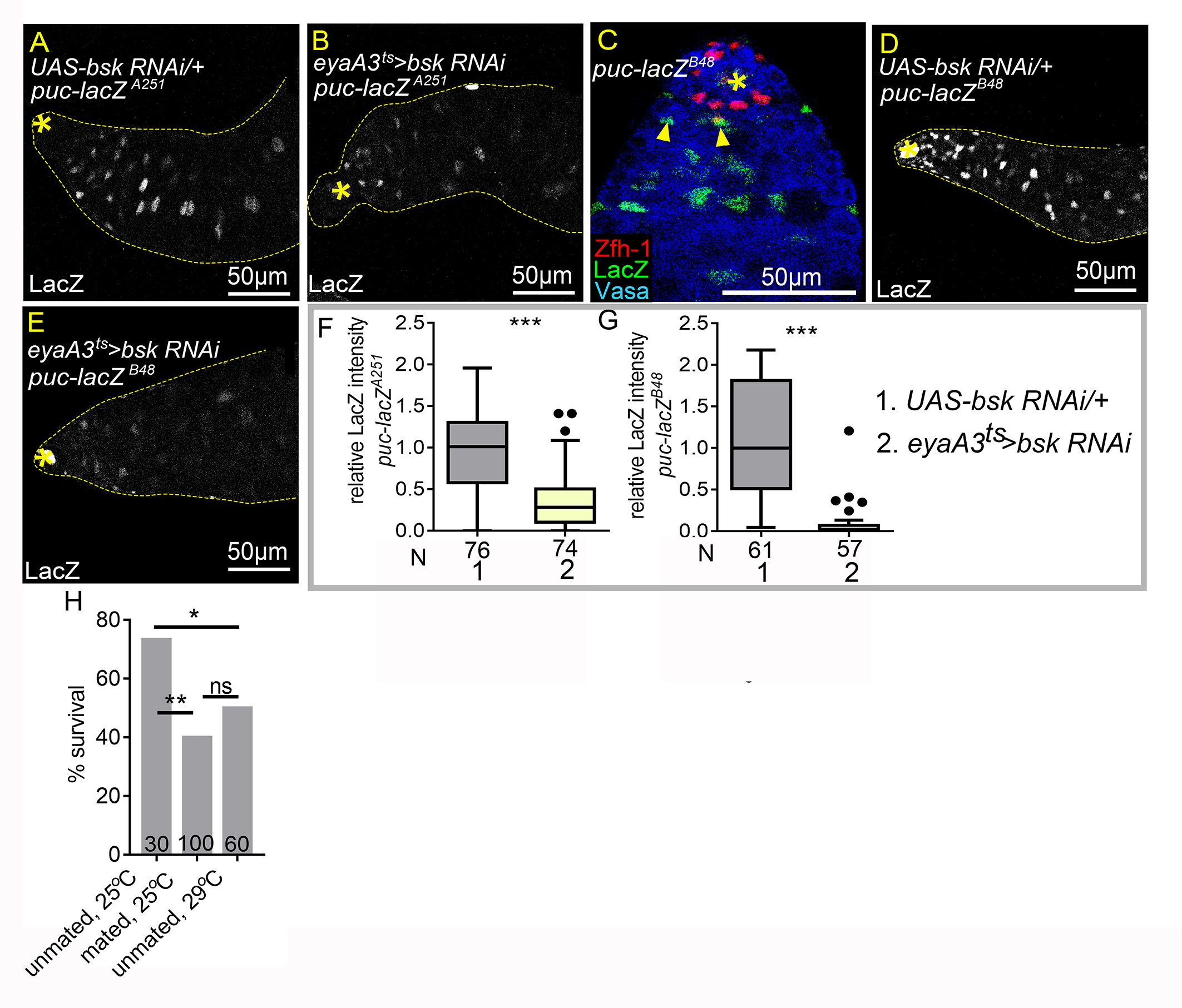

Supplement: S3 Fig — (A, B, D, E) Testes from 1w-old puc-lacZA251 (A and B) and puc-lacZB48 (D and E) males immunostained for β-Galactosidase. LacZ signals were markedly reduced by knockdown of bsk in cyst cells for 7 days. Animals were maintained at 25°C during development. (C) A testis from 3-day-old puc-lacZB48 male immunostained for β-Galactosidase (green), co-stained for Zfh-1 (red) and Vasa (blue). Asterisks mark the hubs. (F and G) Box-and-whisker plots showing normalized LacZ intensity in cyst cells. N: number of the cells scored. Depletion of bsk significantly decreased the LacZ expression intensity in cyst cells. Animals were maintained at 25°C during development. (H) Survival rate of 4w-old puc-lacZA251 males. The unmated 4w-old males a 29°C showed comparable survival rate as mated 4w-old males at 25°C. Numbers of males scored are shown at the bottom of each column. P-values were calculated with Mann-Whitney test in F and G, and Chi-squared test in H. ns: p>0.05, *p<0.05, **p<0.01, and ***p<0.001. Mass-mating (10 vs. 20) and unmated (30 vs. 0) were conducted for H. (TIF) [file pgen.1008062.s004.tif]

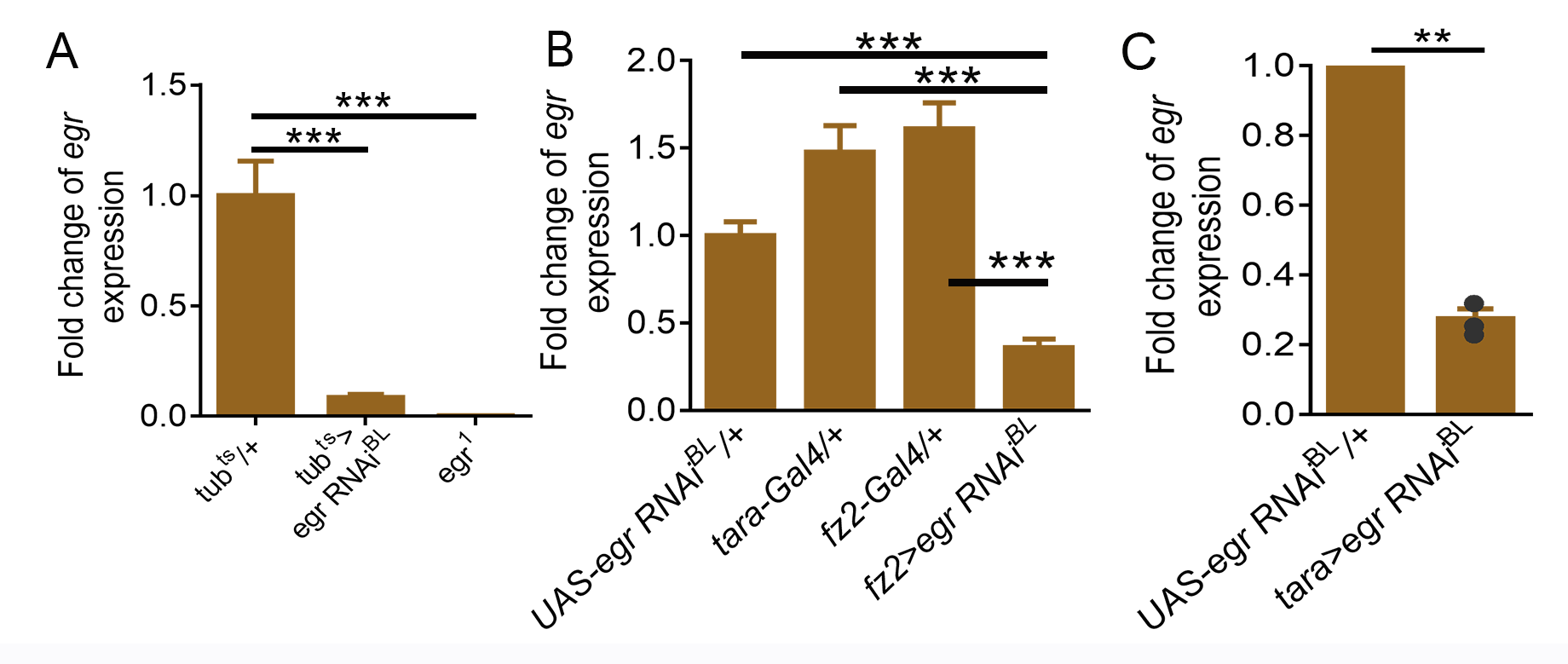

Supplement: S4 Fig — (A) qRT-PCR analysis of egr levels in the 1w-old flies. Error bars represent SD. (B and C) qRT-PCR analysis of egr transcript levels in testes from 1w-old males. The means of egr/rp49 levels from different samples were normalized to that of UAS-egr RNAi testes. Marked decrease of egr mRNA levels was observed in the testes of egr knockdown by fz2-Gal4 (B) and tara-Gal4 (C). (C) N (number of independent biological replicates) = 3. Error bars represent SEM. P values shown in A and B are obtained from student t test. P-value shown in C is obtained from paired t test. *p<0.05, **p<0.01, and ***p<0.001. (TIF) [file pgen.1008062.s005.tif]

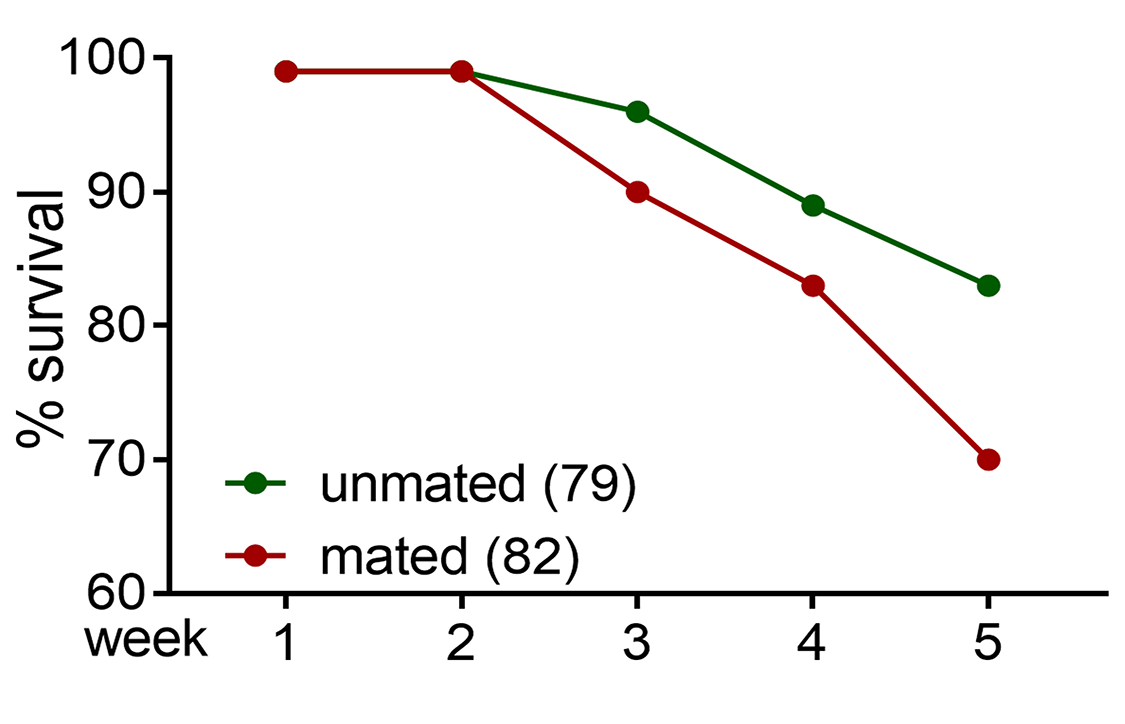

Supplement: S5 Fig — The survival rate of the unmated (1 vs. 0) egr-GFP males at 4w-old was comparable to that of mated 3w-old males (1 vs. 6). The numbers of males are shown in parenthesis. (TIF) [file pgen.1008062.s006.tif]

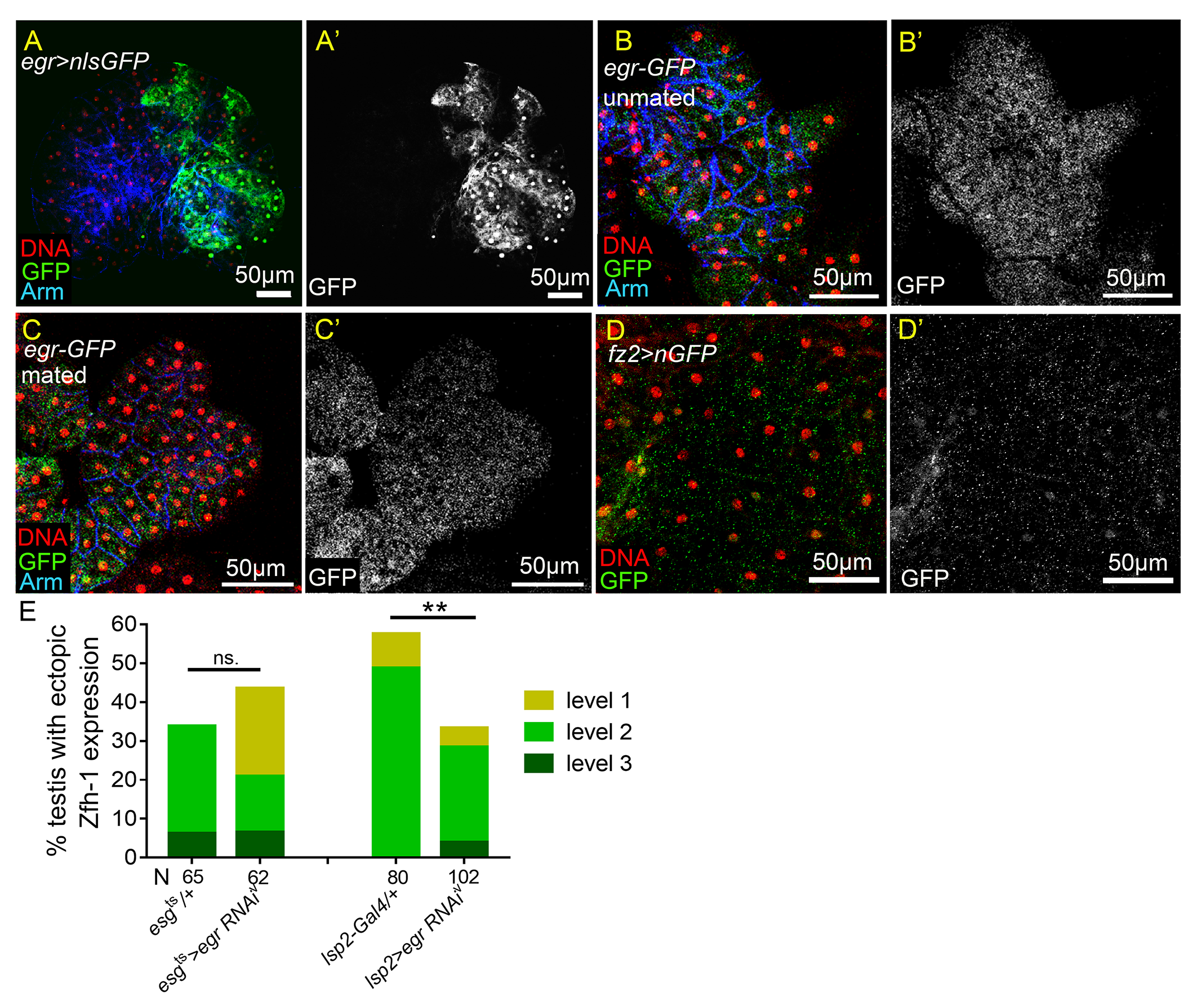

Supplement: S6 Fig — (A and A’) Fat body from 1w-old egr-Gal4>UAS-nGFP male immunostained for GFP (green in A; white in A’), co-stained for Arm (blue) and DNA (red). egr was expressed in adult fat body. (B-C’) Fat bodies from 3w-old egr-GFP males immunostained for GFP (green in B and C; white in B’ and C’), co-stained for DNA (red in B and C) and Arm (blue in B and C). Comparable, low-level GFP expression was observed in fat bodies from unmated (B and B’) and mated (C and C’) males. (D) Fat body from 1w-old fz2-Gal4>UAS-nGFP male immunostained for GFP (green in D and white in D’), co-stained for DNA (red in D). No nuclear GFP signals were detected in fat body. (E) Percentages of testes with ectopic Zfh-1 expression from mated 4w-old males. No or mild suppression was observed by egr knockdown, respectively, in ISC/EB via esgts and in fat body via lsp2-Gal4. P values were calculated with Chi-squared test. ns: p>0.05, *p<0.05, **p<0.01, and ***p<0.001. Mass-mating was conducted in this experiment. (TIF) [file pgen.1008062.s007.tif]
